# A mixed methods evaluation of capturing and sharing practitioner experience for improving local tobacco control strategies

**DOI:** 10.17269/s41997-018-0153-3

**Published:** 2018-11-19

**Authors:** Jennifer Boyko, Barbara Riley, Aneta Abramowicz, Lisa Stockton, Irene Lambraki, John Garcia, Steven Savvaidis, Cynthia Neilson

**Affiliations:** 1 Propel Centre for Population Health Impact, Faculty of Applied Health Sciences, University of Waterloo, 200 University Avenue West, Waterloo, N2L3G1 Canada; 2 School of Public Health and Health Systems, Faculty of Applied Health Sciences, University of Waterloo, Waterloo, Canada; 3 Program Training and Consultation Centre, Toronto, Canada; 4 Canadian Partnership Against Cancer, Toronto, Canada

**Keywords:** Evaluation studies, Evidence-based practice, Public health practice, Documentation, Smoke-free policy, Diffusion of innovation, Études d’évaluation, Pratique factuelle, Pratique en santé publique, Documentation, Politique antitabac, Diffusion de l’innovation

## Abstract

**Objective:**

Practitioner experience is one type of evidence that is used in public health planning and action. Yet, methods for capturing and sharing experience are under-developed. We evaluated the reach, uptake and use of an example of capturing and sharing practitioner experience from tobacco control known as documentation of practice (DoP) reports.

**Methods:**

The participatory, mixed methods approach included the following: a document review to capture data related to the extent and how DoP reports reached the target population; an online survey to assess awareness, use and perceptions about DoP reports; and semi-structured interviews to identify and explore examples of instrumental, conceptual and symbolic use of DoP reports. The samples for the survey and interviews included tobacco control practitioners from public health units in Ontario, Canada.

**Results:**

Seventy-three individuals participated in the survey and 10 were interviewed. Awareness of at least one DoP report was high. The most common way of learning about DoP reports was email. DoP reports focused on policy issues had highest use; these reports were used in conceptual (helped raise awareness), instrumental (directly informed local policy development) and symbolic (confirmed a choice already made) ways. DoP reports may be improved with key messages, shorter development timelines, more relevant topic selection and dissemination to audiences beyond public health.

**Conclusion:**

DoP reports are useful to public health practitioners working in tobacco control within Ontario; refinements to development and dissemination processes will enhance use. Future studies and adaptations of DoP reports could help improve use of practitioner experience as one source of evidence informing public health practice.

**Electronic supplementary material:**

The online version of this article (10.17269/s41997-018-0153-3) contains supplementary material, which is available to authorized users.

## Résumé

**Objectif:**

L’expérience des praticiens est un type d’indice utilisé pour la planification et l’action en santé publique. Cependant, les méthodes pour saisir et partager l’expérience sont sous-développées. Nous avons évalué la portée, la prise et l’utilisation d’un exemple de saisir et partager l’expérience de praticiens en contrôle du tabagisme : des rapports connu sous le nom de la documentation des pratiques (DdP).

**Méthodes:**

L’approche participative et d’une méthodologie mixte comprend une analyse documentaire, un sondage en ligne et des entretiens semi-structurés. L’échantillon comprend des praticiens en contrôle du tabagisme des bureaux de santé publique en Ontario, Canada.

**Résultats:**

Soixante-treize individus ont participé au sondage et 10 ont été interviewés. La connaissance d’au moins un rapport DdP était élevé. La méthode la plus fréquente d’apprendre sur des rapports DdP était par courriel. Les rapports DdP se concentrant sur des problèmes de politiques ont était utilisés le plus; ces rapports ont été utilisé dans des manières conceptuelles (en aidant à sensibiliser), instrumentales (en informant directement le développement des politiques locales) et symboliques (en confirmant un choix déjà pris). Les rapports DdP peuvent être améliorer avec des messages clés; des chronologies de développement plus courtes; une sélection de sujet plus pertinente; et une diffusion aux gens au-delà de la santé publique.

**Conclusion:**

Les rapports DdP sont utiles aux praticiens de la santé publique travaillant en contrôle du tabagisme en Ontario, et le raffinement des processus de développement et de diffusion améliorera leur usage. Des études à venir et des adaptations des rapports DdP pourrait aider à améliorer l’usage de l’expérience des praticiens en tant que source de connaissance scientifique qui informe la pratique en santé publique.

## Introduction

Practitioner experience is a key part of evidence-informed decision-making in public health. Tacit knowledge gained through experience is needed to augment research knowledge so that it is applicable for the local setting (Kothari et al. 2012; Higgins et al. 2011). In public health, practitioner experience is at the centre of the evidence-informed decision-making process because decision-makers must rely on their own and others’ experience to integrate relevant evidence into program planning, implementation and evaluation (NCCMT 2018). Although capturing and sharing practitioner experience can help support its use, little is known about how to do this. In addition, deeper understanding is needed about the actual use of this experience by practitioners in their work (Higgins et al. 2011). This paper describes an initial evaluation of the use of an example of documenting public health experience. The key findings and insights help strengthen our understanding of the use of practitioner experience in public health. Organizations with interest in more explicitly supporting the use of practitioner experience in planning and action can use the results in developing or refining similar approaches.

### Evidence-informed public health

Three main types of evidence used in public health are scientific, practitioner experience and local evidence (NCCMT 2018; Puddy and Wilkins 2011; Dunet et al. 2013). Scientific evidence comes from empirical studies and is often captured through peer-reviewed publications. Local evidence, derived from socio-cultural, socio-economic or governmental conditions within a public health unit’s geographic area, can be found in needs assessments, program evaluations, organizational documents, administrative databases or media reports; it helps decide whether a program or policy fits a particular context (CDCP 2011a). Experiential evidence comes about inductively from practical experience rather than deductively from testing hypotheses developed a priori (CDCP 2011b) and is not formally captured as readily as other types of evidence.

In public health, the experience of practitioners is particularly important because they have knowledge of the local community in which they work, gained through experience taking action under diverse and dynamic conditions (Green 2006). Public health practitioners use their experience to help them account for and consider the complex mix of variables that influence actual practice (CDCP 2011b). This may be done intuitively by drawing on their own tacit knowledge or experience or purposefully by seeking evidence about what has worked and how for others. In this paper, we focus on the use of practitioner experience; evidence derived from working directly in and with communities and that is supportive of local culture and traditions (CDCP 2011b).

### Understanding knowledge use

Our understanding of using practitioner experience for public health planning and action draws on research from the knowledge translation literature. This literature commonly refers to three types of research use: (1) conceptual (diffuse, indirect use to gain understanding, but not necessarily linked to a specific problem or action), (2) instrumental (specific, direct use to solve a problem or take action) and (3) symbolic (persuasive or political use to legitimize, promote or sustain positions, policies or programs already in place) (Straus et al. 2010). Diffusion of innovations theory, for example, is commonly used to understand factors influencing knowledge use (Rogers 2003). These studies include a focus on understanding characteristics of innovations such as systematic reviews of public health interventions that influence the use of the innovation in decision-making (Dobbins et al. 2002). Characteristics of innovations that have been shown to influence use in public health planning and action include perceptions related to the innovation’s relative advantage, complexity and compatibility (Haider and Kreps 2004). Use also requires that intended audiences are aware of knowledge products, read them and understand the relevance to their work (Skinner 2007; Dearing and Kreuter 2010).

Although guidance is available to support the use of research evidence, notably systematic reviews, in public health decision-making, a gap remains in understanding and supporting the use of practitioner experience. In order to address this gap, we evaluated an example of capturing and sharing practitioner experience used by an organization in the field of tobacco control in Ontario, Canada. The example included 16 documentation of practice (DoP) reports produced by the Program Training and Consultation Centre (PTCC) from 2007 to 2016. DoP reports are a knowledge product produced to capture practitioner experience in tobacco control. Supplementary file 1 includes an example of one DoP report. The purpose of the evaluation was to assess the reach, uptake and use of these DoP reports. Specific objectives were to (a) determine the reach and uptake of DoP reports among a group of public health unit practitioners working in tobacco control in Ontario, (b) understand the ways in which these practitioners use DoP reports and perceive their usefulness and (c) identify opportunities to improve DoP reports that will optimize their use and potential for impact among public health practitioners.

### Documentation of practice reports

A logic model that describes the approach used to develop and share DoP reports, as well as intended outcomes, is included in Supplementary file 2. Briefly, each DoP report is focused on a different innovation in tobacco control developed and implemented at the local level by Ontario public health units. Consistent with diffusion of innovations theory, the logic model describes these innovations as practices perceived as new to members of a social system (i.e., public health practitioners working in tobacco control in Ontario) (Rogers 2003). Documenting and sharing innovations in tobacco control practice allow other public health professionals to learn about, adapt and/or adopt the practice. The approach to develop DoP reports used a multiple case study design to understand the innovation. Each case included key informant interviews with public health staff and other stakeholders involved in the specific tobacco control innovation, and a review of relevant documents. The analysis for each DoP report focused on understanding underlying mechanisms that generated key outcomes of interest for each case study and the contextual factors that enabled or constrained these mechanisms from operating (Pawson 2006). Results were compiled into reports and then disseminated through webinars and conference presentations, as well as by field staff who worked with local tobacco control to facilitate the use of tobacco control evidence (PTCC 2015). The intended outcomes of planned development and dissemination activities and subsequent diffusion of DoP reports among practitioners working in tobacco control included the following: awareness of DoP reports and where to access them (short term), use of knowledge gained from DoP reports in local public health initiatives (medium term), and understanding across public health units in Ontario of what tobacco control initiatives work in what contexts (long term).

### Ontario tobacco control context

Tobacco control remains a high priority in public health, and the need for innovative approaches is recognized (McDaniel et al. 2016). This has set the stage for novel strategies that support the use of practitioner experience in decision-making related to tobacco control policy. In Ontario, Canada, the Smoke-Free Ontario (SFO) strategy was introduced in 2004 and included a strong focus on local action through education, programs and policies. Tobacco control area networks (TCANs) support implementation of the SFO strategy by facilitating communication, planning and collaboration among local public health units, non-governmental organizations and other stakeholders. Knowledge development and exchange for the SFO strategy were supported until recently by Ontario’s five tobacco control resource centres. The PTCC (ptcc-cfc.on.ca) was the lead coordinating resource centre and provided training, technical assistance and knowledge exchange opportunities to health professionals working in tobacco control in Ontario. PTCC focused on building the capacity of public health units to deliver evidence-informed tobacco reduction interventions (PTCC 2015). In doing so, public health practitioners benefit from learning about what has worked for other communities and receive support in identifying and using practitioner experience in their local setting.

## Methods

The evaluation team used a participatory approach by engaging a reference group consisting of individuals who were responsible for the production and dissemination of DoP reports. This approach ensured that individuals providing leadership for developing and sharing DoP reports were not also providing leadership for the evaluation, and the evaluation was informed by rich knowledge of the DoP reports, related knowledge translation activities and the tobacco control environment. A mixed methods evaluation design was used to provide a rich understanding of the reach, uptake, use and potential improvements for DoP reports. Consistent with the logic model for the DoP reports (Supplementary file 1), the population of interest was individuals working within Ontario’s 36 public health units and in tobacco control. The evaluation received ethics clearance through a University of Waterloo Research Ethics Committee (#21705).

### Data collection

Data collection took place over a 3-month period in 2016 and involved a document review, online survey and semi-structured interviews.

#### Document review

The extent that DoP reports have *reached* Ontario public health practitioners working in the area of tobacco control was assessed by considering awareness-raising efforts that had taken place from 2011 to 2016. Organizational documents related to DoP reports (e.g., activity tracking logs, evaluations, website analytics) were identified by members of the reference group and provided to the evaluation team. Documents were available from 2011 onward. A data extraction form was used to capture data from these documents related to the extent and how DoP reports reached the target population. These data included the quantities of DoP reports disseminated, as well as the approaches for doing so (e.g., webinars, conference presentations).

#### Online survey

A review of existing tools and methods for assessing reach, uptake and use of knowledge products such as DoP reports was carried out to inform development of the survey. Several questions were adapted from a previously developed tool to measure knowledge translation outcomes of single interventions (e.g., awareness or use of a best practice guideline) (Skinner 2007) by modifying wording, response options and how questions fit together conceptually. It was necessary that our survey be appropriate for public health practitioners, on-line use, and assessment of multiple interventions (i.e., all DoP reports that have been developed and disseminated). The final survey consisted of five parts: (a) awareness of DoP reports, (b) use of DoP reports, (c) perceptions about DoP reports, (d) improvements and (e) information about the participant.

Awareness of DoP reports and how individuals became aware of them were indicators of the *reach* of DoP reports within the target population. *Uptake* of DoP reports was assessed by determining the extent that individuals had read but not used specific DoP reports. *Use* of DoP reports was assessed through questions pertaining to type of use and factors that may influence use. Survey questions probed conceptual, instrumental, and symbolic knowledge use. Potential for *improvement* was assessed through questions related to specific characteristics of DoP reports, such as ease of understanding, visual presentation and relevance of topics.

A convenience sample of 211 potential survey participants was identified from a PTCC database. The sample consisted of public health practitioners who had been involved in PTCC community of practice (CoP) activities. This sample was considered sufficient for an initial evaluation of DoP reports. Data collection was performed using an online survey tool (Propel Survey Solutions). An email invitation that included a web link to complete the survey was sent to all potential participants. Reminder emails were sent weekly for 4 weeks to those who did not respond (excluding those who declined participation). Participants were able to visit their unique survey link as often as they wanted before submitting their responses.

#### Semi-structured interviews

An interview guide was devised in order to identify and explore examples of instrumental, conceptual and symbolic use of DoP reports, as well as opportunities for enhancing reach, uptake and use of them. A sample of 30 potential interview participants was identified from survey respondents who indicated their willingness to participate in a follow-up interview and from the reference group. The sample included individuals with varying types of experience using DoP reports, as well as individual, organizational and community characteristics. An email invitation was sent to all potential participants. One team member worked with each individual who indicated willingness to participate to schedule an agreeable time. Interviews were up to 60 min in length, digitally recorded and transcribed. Each interviewee was given the opportunity to review their transcript for accuracy.

### Analysis

Quantitative data from the survey and document review were compiled and analyzed using descriptive statistics. Written responses from survey questions were summarized narratively. Qualitative analysis involved coding interview transcripts using both inductive and deductive approaches. An initial coding framework was developed based on the interview guide (deductive) and then enriched with refinements and new codes that emerged from the interview data (inductive). Two members of the research team met frequently to discuss and reach agreement on emerging themes, as well as enhancements to the coding framework. All themes were also compared on an ongoing basis, and similar themes grouped together. One team member completed all coding, and a second team member reviewed all coding for consistency, accuracy and completeness. Upon completion of coding all transcripts, a thematic analysis of all codes was carried out to identify and articulate key themes among the data. Memos were used throughout the analysis to track and guide emerging ideas and thought processes. Qualitative analysis (including data storage, indexing, searching and coding) was undertaken using NVivo 11 software.

Quantitative and qualitative results were compared, contrasted and integrated in order to identify overarching evaluation findings. The key findings and supporting data were presented at a meeting with a select group of individuals, including members of the reference group and others with knowledge of DoP reports (PTCC field staff, study coordinators). The purpose of the meeting was to interpret and contextualize key findings and explore potential implications for public health practitioners who work in tobacco control.

## Results

The survey response rate was 35% (*n* = 73). Survey participants represented a variety of public health roles (e.g., health promoter, tobacco control coordinator), and the majority devoted more than half of their work time to tobacco control issues (72%) and had worked in the area of tobacco control for more than 8 years (53%). The final survey sample also included representation from each TCAN in Ontario and was equally representative of public health units that served primarily rural and urban populations.

The interview response rate was 32% (*n* = 10). Interview and survey samples reflected similar diversity in terms of individual, organizational and community characteristics. The proportions of participants who indicated experience with the process of developing DoP reports were high among survey and interview participants; 55% of survey respondents and 40% of interview participants had been involved in the development of one or more DoP reports. These proportions are not likely representative of the true proportion within the target population. Additional characteristics of the survey and interview sample are described in Table 1.

**Table 1:** Participant characteristics

	Survey % (*n* = 73)	Interviews % (*n* = 10)
PHU role		
Health promoter	23	30
Public health nurse	23	20
Tobacco control manager	21	10
Other	13	40
Tobacco control coordinator	12	20
Tobacco enforcement officer	7	–
Director	2	–
Time spent on tobacco control		
0–25%	13	–
26–50%	15	–
51–75%	20	20
76–100%	53	80
Years in tobacco control		
Less than 1 year	2	–
1–4 years	35	20
5–7 years	10	10
8–10 years	22	40
More than 10 years	32	30
Involvement in a CoP		
Attending CoP meetings	56	80
Participating in discussions on Ning	21	20
Sharing/accessing resources on Ning	27	4
Connecting directly with other CoP members	34	80
Not involved	16	–

*PHU* public health unit, *CoPs* communities of practice, *Ning* online community building platform

### Comparison of reach, uptake and use

Table 2 includes a summary of survey data related to reach (awareness), uptake (read but not used) and use for each of the DoP reports. Comparison of the reach, uptake and use of specific DoP reports offers insight about the extent to which diffusion had taken place. Among those who responded to the survey, 85% were aware of at least one of the DoP reports. Awareness of specific DoP reports was highest (41%) for the most recent and lowest (11%) for the oldest. The DoP reports with the highest uptake included the most recent one, as well as two focused on policy-oriented topics (e.g., smoke-free housing policy). The DoP report with the lowest uptake (4%) was focused on a topic less relevant to a wide audience. The DoP reports with the highest indication of use focused on policy issues, including smoke-free outdoor spaces (45%) and smoke-free housing (40%).

**Table 2:** Reach, uptake and use for each DoP report

	Reach: aware of this DoP report	Uptake: read this DoP report	Use: used this DoP report
DoP reports and year developed^*^	% (*n*)		
Reaching priority populations who experience barriers to smoking cessation supports (2016)	41 (24)	9 (5)	19 (11)
Building local capacity for smoking cessation supports (2016)	36 (21)	19 (11)	15 (9)
The Development of the Indoor Smoke-Free Space Movement (2016)	29 (17)	12 (7)	20 (12)
Creating Smoke-Free Hospitals in Ontario (2013)	34 (20)	19 (11)	27 (16)
Creating Smoke-Free Spaces: The Development of Smoke-Free Outdoor Space By-Laws (2013)	23 (13)	9 (5)	45 (25)
Promoting Smoke-Free Multi-Unit Dwellings in the Peterborough County-City Region (2012)	24 (14)	16 (9)	28 (6)
Choose To Be... Smoke-Free: Peterborough County-City Health Unit’s Woman-Centred Program (2012)	14 (8)	7 (4)	16 (9)
Recruiting Young Adults into Focus Groups: Findings from the No Butts About It Project (2012)	21 (12)	4 (2)	7 (4)
The Development of the Central West Tobacco Control Area Network’s System of Local Tobacco Cessation Communities of Practice (2011)	19 (11)	5 (3)	17 (10)
Development of the Region of Waterloo’s Minimal Contact Intervention for Tobacco Cessation Policy: Key Success Factors and Lessons Learned from Practice (2011)	16 (9)	9 (5)	21 (12)
The Development of a Smoke-Free Housing Policy in the Region of Waterloo: Key Success Factors and Lessons Learned from Practice (2010)	19 (11)	21 (12)	40 (23)
The Development and Promotion of Guelph Soccer’s Tobacco-Free Policy: Key Success Factors and Lessons Learned from Practice (2010)	21 (12)	11 (6)	9 (5)
Partnerships Developed Between Ontario’s Local Public Health Agencies (LPHAs) & Ontario Hockey League (OHL) Teams: Key Success Factors & Lessons Learned from Practice (2010)	23 (13)	9 (5)	11 (6)
Smokers’ Section: Supporting Youth who Use Tobacco in the Ottawa Area Through an Innovative, High School-Based Triage Program (2008)	14 (8)	9 (5)	11 (6)
Legislation & Implementation of Collingwood By-laws 00-36 and 05-36: Smoking Ban for Playgrounds & Playing Fields (2008)	18 (10)	7 (4)	35 (20)
Tobacco Treatment for New Canadians in Waterloo Region: Lessons Learned (2007)	11 (6)	7 (4)	4 (2)

^*^Respondents for each DoP report ranged from *n* = 57 to *n* = 59

### Awareness and dissemination efforts

Awareness was created through planned dissemination efforts. Details gathered from the document review indicated that dissemination included the following: PTCC hosted webinars that featured different DoP reports (*n* = 7), conference presentations (*n* = 16) and presentations made as part of a CoP (*n* = 16). Ways that respondents became aware of the DoP reports are presented in Table 3. The most common means of awareness were emails from PTCC (61%) and the PTCC website (48%), while the least common were knowledge exchange forums (31%), CoP meetings/discussions/activities (32%) and PTCC staff (32%). Respondents that selected “other” noted that they became aware of DoP reports by colleagues in the public health community or from being involved in developing one. Interviews revealed that the public health practitioners shared DoP reports with a variety of partners, including hospitals and healthcare providers, housing staff and workplace representatives. Sharing was based on information needs and involved sharing a DoP report in its entirety, either in physical or digital format, or selectively sharing pieces of information contained in a single report for a specific purpose.

**Table 3:** Ways that respondents became aware of the DoP reports

Response options	% (*n*)
Email from PTCC	61 (38)
PTCC website	48 (30)
CoP collaborative website	36 (22)
PTCC provincial webinar	34 (21)
CoP meetings/discussions/activities	32 (20)
PTCC staff	32 (20)
PTCC knowledge exchange forum	31 (19)
A workshop delivered by PTCC	23 (14)
Other	19 (12)
Do not recall	8 (5)
Other presentations	6 (4)

Respondents could select more than one response option. Thus, percentages do not add up to 100%

The interview findings were consistent with the survey results and provide further insight into dissemination efforts that worked best or could be improved to raise awareness. For example, one individual specifically noted emails as a key way they learned about DoP reports, but also noted that other dissemination efforts were helpful: “I’m aware of them, my colleagues are aware of them because we get emails … and we’re involved with the TCANs….you see it multiple times ...you get the notifications about the webinars or that they’re available”. Another individual indicated that emails effectively raised awareness about new DoP reports but were not “catchy enough” to persuade reading through DoP reports. Strategies suggested for fostering uptake included improving visual appeal (e.g., more graphics), informally encouraging colleagues that DoP reports are “worth the read” and continuing to share DoP reports with audiences beyond public health units.

### Types of use

Measures of conceptual, instrumental and symbolic use of DoP reports are presented in Table 4. Agreement to the eight statements pertaining to conceptual use ranged from 84% (“The DoP(s) got me thinking differently about a particular approach to practice”) to 35% (“I cited the DoP(s) in my reports or documents”). Agreement to each of the four statements pertaining to instrumental knowledge use was 72% or higher. Agreement to the single statement pertaining to symbolic use was 82%. Table 5 includes a summary of questions pertaining to factors that influence use of DoP reports. More than half of respondents indicated agreement with each statement, with the only exception being “I cited the DoP(s) in my reports or documents”, which had 35% agreement.

**Table 4:** Measures of conceptual, instrumental and symbolic uses of DoP reports

Measures of different types of use*	Agree^†^ % (*n*)	Disagree^‡^ % (*n*)	Do not recall % (*n*)
Instrumental:			
I adopted a new practice based on guidance in the DoP(s).	72 (39)	15 (8)	13 (7)
I modified an existing practice(s) based on guidance in the DoP(s).	76 (41)	9 (5)	15 (8)
I plan to adopt or modify a practice(s) outlined in the DoP(s).	73 (40)	13 (7)	15 (8)
The DoP(s) prompted me to develop one or more new partnerships/collaborations for my work in tobacco control.	72 (39)	15 (8)	13 (7)
Conceptual:			
I encouraged or persuaded a colleague(s) to adopt a practice(s) outlined in the DoP(s).	54 (29)	28 (15)	19 (10)
I have discussed the DoP(s) with colleagues in my health unit.	76 (42)	15 (8)	9 (5)
I plan to discuss the DoP(s) with colleagues in my health unit.	76 (41)	13 (7)	11 (6)
I have discussed the DoP(s) with colleague(s) outside of my health unit.	51 (28)	29 (16)	20 (11)
I plan to discuss the DoP(s) with colleague(s) outside of my health unit.	50 (27)	39 (21)	11 (6)
I cited the DoP(s) in my reports or documents.	35 (19)	40 (22)	26 (14)
I plan to cite the DoP(s) in my reports or documents.	47 (25)	42 (22)	11 (6)
The DoP(s) got me thinking differently about a particular approach to practice.	84 (46)	7 (4)	9 (5)
Symbolic/strategic:			
The DoP(s) served to confirm choices already made in my work.	82 (44)	7 (4)	11 (6)

*Statements are in relation to all DoP reports that respondents were aware of and/or used; respondents to each statement ranged from *n* = 53 to *n* = 55

^†^Includes Agree and Strongly Agree

^‡^Includes Disagree and Strongly Disagree

**Table 5:** Factors that influence DoP report use

Statements about factors that influence DoP report use*	Agree % (*n*)	Disagree % (*n*)	Do not recall % (*n*)
The DoP(s) that I am aware of and/or used influenced my thinking or work because the practice insights outlined therein:			
Were supported by sufficient research evidence to demonstrate that the practice would be effective or successful.	81 (44)	4 (2)	15 (8)
Could lead to greater impact than previous practices.	74 (40)	4 (2)	22 (12)
Were innovative and leading edge.	69 (37)	9 (5)	22 (12)
Could be implemented on a small scale or limited basis to determine its advantages or disadvantages.	67 (36)	11 (6)	22 (12)
Required less time and/or effort than previous practices.	33 (18)	28 (15)	39 (21)
Were less costly than previous practices.	22 (12)	22 (12)	56 (30)
The DoP(s) that I am aware of and/or used influenced my thinking or work because I, in my professional role:			
Learned new information about how to implement a practice(s) successfully.	87 (45)	2 (1)	12 (6)
Learned new information about the effectiveness of a practice(s).	85 (44)	6 (3)	10 (5)
Have values and beliefs consistent with the practice.	85 (44)	0 (0)	15 (8)
Had enough decision-making authority to decide to adopt the practice.	62 (32)	27 (14)	12 (6)
Had sufficient time to adopt and implement the practice.	59 (30)	18 (9)	24 (12)
Was able to prove to my supervisor that this was an important practice to adopt.	49 (25)	25 (13)	25 (13)
The DoP reports that I am aware of and/or used influenced my thinking or work because the organization or community I work with:			
Was in a location or setting where adopting or implementing the DoP practice insights made sense.	73 (37)	8 (4)	20 (10)
Has values consistent with the DoP practice insights.	71 (36)	6 (3)	24 (12)
Had enough collaboration or potential for networking with other organizations to be able to adopt and implement the DoP practice insights.	67 (34)	8 (4)	25 (13)
Was facing a relevant challenge that could be addressed by DoP practice insights.	65 (33)	8 (4)	27 (14)
Was an appropriate size (i.e., not too big or small) to adopt DoP practice insights.	60 (30)	10 (5)	30 (15)
Had policies or procedures that fit with or supported the DoP practice insights.	59 (30)	8 (4)	33 (17)
Had enough resources (i.e., staff, financial) to adopt the DoP practice insights.	53 (27)	20 (10)	27 (14)
Was not already doing what the DoP(s) was suggesting.	53 (27)	25 (13)	22 (11)
In my opinion, the DoP(s) are:			
Credible in terms of their source (i.e., PTCC and Propel).	95 (52)	0 (0)	5 (3)
Supported by theory and appropriate literature.	91 (50)	0 (0)	9 (5)
Easy to understand.	89 (49)	2 (1)	9 (5)
Realistic in terms of recommendations and implications.	87 (48)	2 (1)	11 (6)
Focused on timely and relevant topics.	87 (48)	7 (4)	5 (3)
Shared with tobacco control practitioners through appropriate channels.	85 (47)	7 (4)	7 (4)
Presented in an appealing way (graphics, colour, packaging).	76 (42)	9 (5)	15 (8)

*Respondents to each statement ranged from *n* = 51 to *n* = 55

The interviews provided further insight into use of DoP reports. A theme related to conceptual knowledge use was that DoP reports helped raise awareness about priority tobacco control issues. As one individual described: “…what is most useful is that [the community] learn they’re not alone... it’s helpful and comforting for them to know that others have done it.” Another theme was that knowledge gained from DoP reports was used to address tobacco control problems in indirect ways, such as building a case for action (e.g., to inform a recommendation to municipal council regarding policies surrounding outdoor smoking legislation), developing work plans for public health unit initiatives and informing organizational plans by helping to establish priority areas.

A key theme related to instrumental knowledge use was that DoP reports have been used to directly inform and establish local policies on tobacco control. For example, DoP reports have been helpful in guiding the legal process when working to pass legislation or writing by-laws. Consulting and supporting community partners was also a theme related to instrumental use. Those interviewed provided examples of actions informed by DoP reports, such as supporting communities with policy development (e.g., establishing a Minimal Contact Intervention policy at a partner hospital), implementation (e.g., assisting housing boards to enact smoke-free housing policies) and enforcement (e.g., supporting a hospital to enforce an existing smoke-free policy).

### Factors that influence use

Overall, there was high agreement to all survey statements about characteristics of DoP reports that may support use. The characteristics with the highest agreement were the following: “credible in terms of their source” (95%), “supported by theory and appropriate literature” (91%) and “easy to understand” (89%). The lowest rated characteristic was “presented in an appealing way (graphics, colour, packaging)” (76%).

Suggestions for improving use of DoP reports identified from the interviews and survey responses focused on format, dissemination, topic/case selection and the development process. Improving the format of DoP reports was a prominent overall theme. Specifically, suggestions were made related to including a summary of content and key lessons: “The summaries to me are things that can be shared with partners…rather than send an entire documentation, the quick one- or two-pagers…are very useful in terms of sharing”. Other suggestions included making DoP reports more captivating, especially at first glance, via visual enhancement (e.g., infographics) and/or interactive components (e.g., short videos), using consistent formatting across DoP reports and including more detail and resources to support project implementation (e.g., sample forms, staffing requirements, project costs).

Dissemination was another prominent theme, including to audiences beyond public health. For example, one participant noted, “wider sharing of these practices outside of the [public health] sector might allow for great coalition and community based work”. Some survey respondents indicated a lack of knowledge about where to access DoP reports; others suggested more widespread and systematic distribution. Interviewees suggested greater promotion at the TCAN levels (e.g., adding DoP-related discussion to the agenda of all TCAN meetings; ensuring new tobacco control public health practitioners are aware of DoP reports and where to access them). Improving email distribution was another suggestion (e.g., making emails related to DoP reports stand out; ensuring public health practitioners new to tobacco control belong to the PTCC listserv).

Another common suggestion was improving the DoP development process. Suggestions included a faster development process with greater alignment with local public health priorities and updating existing DoP reports with new case examples or evidence. Other suggestions focused on improving practitioner engagement in the development process through more opportunities to be involved in the selection of priority topics or design of DoP reports. A few individuals felt that DoP reports would be more relevant if they featured greater variety of cases and contexts.

## Discussion

DoP reports are a knowledge product produced in order to capture and share practitioner experience related to tobacco control. This evaluation focused on reach, uptake and use of DoP reports among a select group of public health practitioners working in tobacco control in Ontario, Canada. The evaluation findings demonstrate that many of the public health practitioners are aware of DoP reports and have used them to inform policy actions or support community partners with policy-related information. Consistencies between the evaluation results and knowledge translation in public health literature related to knowledge use (Valaitis et al. 2016; Dobbins et al. 2009; Dearing and Kreuter 2010) help to highlight key findings, new thinking about factors to consider in development and dissemination processes, as well as future research priorities.

Three key findings are apparent from the results. First, awareness and use of DoP reports were consistent with how diffusion of an innovation is theorized to occur within a social system; PTCC used dissemination strategies including webinars, emails, presentations and technical field staff in order to communicate new DoP reports to knowledge users. These active and purposeful strategies initiated the diffusion process (Greenhalgh et al. 2004; Rogers 2003). The most recent DoP reports had highest awareness, suggesting they were in an early stage of diffusion among the public health practitioners. The process of passive diffusion (Greenhalgh et al. 2004) takes time, which may explain the high use of older policy-oriented DoP reports. The topics of earlier DoP reports may have emerged over time as priorities across many public health units, or practitioners had more time to engage in informal communication with peers in their network of public health practitioners working in the tobacco control system. We know that social systems are essential to the diffusion of an innovation. Our evaluation demonstrates the value of having common goals among members of social networks who share knowledge informally (Valaitis et al. 2016). The public health practitioners who participated in the evaluation shared goals for reducing the impact of tobacco use, which facilitated communication over time among them.

The second key finding from the evaluation is that DoP report use was supported by characteristics of the tobacco control innovation described therein. For example, the DoP reports with highest use addressed issues with high relevance to public health practitioners; these reports coincided with policy priorities in Ontario (i.e., tobacco by-law development and implementation). Another characteristic that supported use was perceived complexity of the practice insights outlined in the DoP reports. Reports were considered easy to understand by the public health practitioners. This may be a function of the nature of practitioner experience captured in DoP reports or the “packaging” of the report itself (graphics, colour, layout). Perceived complexity can be reduced by practical experience (Greenhalgh et al. 2004), which is the type of knowledge captured in DoP reports. Another characteristic that supports use is compatibility. Innovations that are compatible with organizational or professional norms, values and ways of working are more readily adopted (Greenhalgh et al. 2004). Public health practitioners indicated that DoP reports influenced their thinking or work because the practitioner experience captured reflected values and beliefs consistent with their own practice, as well as that of their organization.

Our third key finding is that characteristics of the local policy context may have influenced the type of use for DoP reports focused on tobacco control policies. Communities can use DoP reports in ways that suit their stage of readiness for policy change. As Greenhalgh et al. (2004) point out, an organization may be amenable to an innovation in general but not ready to take action to implement it. In the context of the current study, this could mean a public health unit is aware of a particular DoP report (including the policy innovation it represents) but not ready to use the knowledge. In such cases, the practitioner experience within DoP reports can be used conceptually to “set the stage for action” by building a case for action or raising awareness about the issue. An external mandate may increase an organization’s motivation to act and use knowledge in more direct or instrumental ways (Greenhalgh et al. 2004). This may explain why DoP reports with the highest indications of use aligned with government directives to implement policies related to smoke-free outdoor spaces and smoke-free housing. Public health practitioners can use the insights gained from a DoP to adapt a policy development process to their local community, leading to a higher chance of successful implementation over communities that routinize their implementation process (Greenhalgh et al. 2004). Symbolic use of DoP reports can be helpful in the policy evaluation and assessment phase when support for a particular policy already in place is needed to ensure continued support or funding. Overall, use of DoP reports focused on tobacco control policies can vary depending on the current policy context.

### Implications for public health practice

We highlight three implications of the evaluation findings for public health practitioners. First, it may be possible to increase awareness, uptake and use of knowledge products, such as DoP reports, through improvements relating to format, dissemination, topic and case selection and the development process. Additional potential improvements may include a faster development process in order to meet priority policy needs, and updating existing knowledge products to keep them current. Second, given that the findings support the notion that innovations in knowledge translation must undergo a process of diffusion prior to uptake and use, specific knowledge translation activities (e.g., peer-to-peer learning via communities of practice) could be explored to determine the most effective activities for spreading knowledge product use among public health practitioners. There is currently limited guidance in the literature to do so, especially tailoring to specific characteristics of the knowledge or innovation to be transferred, intended knowledge users and organizational or community context (LaRocca et al. 2012; Grimshaw et al. 2006). Third, a follow-up evaluation could contribute understanding about the effects of DoP reports on local tobacco control strategies and outcomes and potential for using or adapting this particular knowledge product to capture and share practitioner experience in other priority public health areas. Ultimately, a model to support planning and action for documenting and sharing practitioner experience may be developed (Tiffany and Lutjens 1998; Dearing and Kreuter 2010).

### Limitations

The most notable limitation is that the favourable results on awareness and use of the DoP reports, alongside many and fundamental suggestions for DoP report improvements, are somewhat difficult to reconcile. One possible explanation is the novelty of DoP reports as a source of evidence in public health. This could result in favourable results on use of DoP reports, since experiential evidence fills an important knowledge gap and practitioners appreciate this new type of information. The newness of the type of evidence could also explain extensive suggestions for improvements. Another limitation relates to the representativeness of the study sample, which was likely a more engaged and discerning group than the total population of potential DoP report users. It is possible that the high awareness of DoP reports found is attributable to the convenience sample (i.e., PTCC compiled the survey sample from lists of community practice members) and self-selection of experienced DoP report users. A better understanding of those who did not participate in the survey and interviews would help ensure the evaluation is representative of the target population. Study measures are another limitation. The evaluation measured the frequency of use of DoP reports overall, since the response burden was considered too high to assess reach, uptake and use with individual DoP reports. Survey questions pertaining to perceptions about specific characteristics of DoP reports were intended to help identify opportunities for improvement. However, there was high agreement to all these statements. Interviews provided more helpful data related to improvements.

## Conclusion

DoP reports are a knowledge translation activity intended to support a more coherent understanding of what works and for whom in relation to tobacco control, so that public health units can appropriately respond to local issues. The approach used by one organization for capturing and sharing practitioner experience is useful to public health practitioners working in the area of tobacco control within Ontario. This evaluation demonstrates that various factors support use of DoP reports; however, the relative advantage of these factors is unknown. Further, understanding is required about the processes (e.g., end-user involvement in the development process) and conditions (e.g., alignment of topics with policy priorities) that encourage or dissuade the use of knowledge products such as DoP reports.

## Electronic supplementary material

ESM 1 (PDF 1.03 mb)

ESM 2 (PDF 225 kb)
