# Supplementary material for: A mixed methods evaluation of capturing and sharing practitioner experience for improving local tobacco control strategies
Source: Can J Public Health. 2018 Nov 19;110(1):103–13. doi: 10.17269/s41997-018-0153-3 (PMC6335370; doi:10.17269/s41997-018-0153-3)
Supplement: Supplementary file 1 — (PDF 1.03 mb) [file 41997_2018_153_MOESM1_ESM.pdf]

CREATING SMOKE-FREE SPACES: THE DEVELOPMENT OF SMOKE-FREE OUTDOOR SPACE BY-LAWS

A DOCUMENTATION OF PRACTICE REPORT

MARCH 2013

The Program Training and Consultation Centre conducts applied research in partnership with the Propel Centre for Population Health Impact at the University of Waterloo.

Suggested citation

McCammon-Tripp, L., Hakvoort, M., Shields, T., Czoli, C., Hoekstra, B., Lambraki, I., & Garcia, J. (2013). *Creating smoke-free spaces: The development of smoke-free outdoor space by-laws.* Toronto, Ontario: Program Training and Consultation Centre and the Propel Centre for Population Health Impact, University of Waterloo.

Prepared by

L. McCammon-Tripp[1], M. Hakvoort[1], T. Shields[1], C. Czoli[1], B. Hoekstra[1], I. Lambraki[1], J. Garcia[1, 2, 3]

[1]Propel Centre for Population Health Impact
[2]School of Public Health and Health Systems, University of Waterloo
[3]Ontario Tobacco Research Unit

For further information regarding this report, please contact:

Dr. John Garcia
School of Public Health and Health Systems
University of Waterloo
200 University Avenue West
Waterloo, ON
N2L 3G1

Telephone: 519-888-4567 x 35516

E-mail: john.garcia@uwaterloo.ca

Program Training and Consultation Centre

The Program Training and Consultation Centre (PTCC), founded in 1993, is a resource centre of the Government of Ontario's Smoke-Free Ontario Strategy (SFO). PTCC acts as a knowledge broker between local public health departments, the research community, and government. Its strategic priorities are to:

- Build the capacity of Ontario's 36 public health departments to plan and implement evidence-based tobacco control programs
- Support moving evidence into action
- Strengthen program development and applied research efforts
- Build system capacity to support the Smoke-free Ontario Strategy renewal

Funded through Public Health Ontario, PTCC is a partnership between Cancer Care Ontario and the Propel Centre for Population Health Impact at the University of Waterloo. Propel supports PTCC's priorities through translating research evidence for practitioners, documenting practice-based knowledge and experiences, and conducting applied intervention research.

Propel Centre for Population Health Impact

Propel is a collaborative enterprise that conducts research, evaluation and knowledge exchange to move evidence into action, accelerating improvements in the health of populations in Canada and around the world.

Acknowledgements

The authors would like to acknowledge all of the individuals who participated in this study as key informants. Their willingness to share their knowledge of smoke-free outdoor space by-laws is much appreciated, and will be valued by those who learn from their experiences. The authors would specifically like to acknowledge:

- Kevin McDonald and Heidi McGuire from the City of Hamilton Public Health Services
- Department of Cemeteries and Parks, City of Hamilton
- Pippa Beck and colleagues from the Ottawa Council on Smoking or Health
- Simcoe Muskoka District Health Unit
- Hilda Chow, Krista Oswald and colleagues from Ottawa Public Health

Summary of Findings

Ontario has a strong history of creating smoke-free spaces to improve Ontarians' health outcomes. The first phase of by-law development focused on the creation of smoke-free indoor spaces. This phase began with municipalities and regions creating local level by-laws which ultimately paved the way for the passage of the *Smoke-Free Ontario Act* (SFOA) at the provincial level. Currently, local municipalities are again taking the lead in the creation of smoke-free spaces, this time with a focus on smoke-free outdoor spaces including parks, playgrounds, municipal property, and patios. Outdoor smoking restrictions not only reduce exposure to second-hand smoke (SHS), they may also decrease the risk of fire, reduce tobacco product related litter, and protect community members from nuisances (Bloch & Shopland, 2000). They may also play a role in changing social norms related to tobacco-use (Alesci, Forester & Blaine, 2003). While many municipalities have adopted by-laws creating smoke-free outdoor spaces, there is great variation in the comprehensiveness of the by-laws developed. This documentation of practice project explored the contexts and mechanisms associated with the development of by-laws with high regulatory strength in three Ontario communities.

Methods

Taking a realist approach (Maxwell, 2004; Pawson & Tilley, 1994), the study aimed to understand what works, for whom, and under what conditions to develop smoke-free outdoor space by-laws. A multiple-case study design (Stake, 2006; Yin, 2009) was used to investigate this topic. The following municipalities with comprehensive smoke-free outdoor space by-laws were selected as cases for the study: Barrie, Hamilton, and Ottawa. Key informant interviews were conducted with individuals involved in the by-law development process and documents were collected. Within and cross-case analyses were completed. This summary highlights the cross-case results. Individual case results are available in Appendices A to C.

Results

The policy process undertaken in the three cases fits within the model of policy development conceptualized by Kingdon (2003) who suggests that within politics there are three main streams – the problem, policy, and political streams. These streams are largely independent, but when they converge a policy window opens and there is opportunity for policy development. Given that many different issues are of interest to decision makers at any given time, it is critical to ***create and take advantage of policy windows*** to create action in an area of interest***.***

Policy entrepreneurs individuals with *"a willingness to invest their resources – time, energy, reputation and sometimes money – in the hope of a future return"* (Kingdon, 2003, p. 122) played a critical role in all three cases. The importance of these policy entrepreneurs in the development and implementation of smoke-free outdoor space by-laws cannot be minimized. Across the cases, various individuals took on the role of policy entrepreneur including: public health practitioners, City Councillors, community advocates, and youth. The fact that policy entrepreneurs from different backgrounds initiated the policy development process in the three cases suggests that any individual with an interest in this area can be the driver of change.

Policy entrepreneurs across the three cases used similar mechanisms to create policy windows and move by-law development and implementation forward. These are described in the next section.

Mechanisms related to by-law development

The following mechanisms were highlighted as playing a critical role in the by-law development process. Aspects of these mechanisms may also apply to other phases of the by-law process, such as implementation, and should be considered throughout.

Understanding the problem – drawing on scientific and practice-based evidence - Across the three cases it was clear that decision makers and communities understood the dangers of SHS indoors, but there was less awareness of the issue in the outdoor setting. Sharing the scientific evidence available to support the need for smoke-free outdoor

spaces through reports, media releases, and events, primary research and expert testimony was one way policy entrepreneurs shed light on the problem. Highlighting practice-based evidence – the experiences of other municipalities who have addressed outdoor tobacco use – was also valuable for drawing attention to the issue.

Build on past successes: Moving from indoor to outdoor smoke-free spaces - Like many municipalities across the province, each of the three cases had previously adopted smoke-free indoor space by-laws. The cases studied used this experience to their advantage and built on the lessons learned (i.e., both the successes and lessons on how to do things better) from this past work.

Allow time for Council and the community to "soften up" on the issue - Kingdon (2003) asserts that it is extremely beneficial to ensure that decision makers and the public are aware of a proposed solution to a problem so when a policy window opens there is already readiness for by-law development. This is referred to as the "softening up" process. Various activities were undertaken to allow for this softening up to occur in the three cases including: educating the public and decision-makers about changing social norms related to tobacco use within the community and more broadly, highlighting the need to expand the number of places that are smoke-free, beginning with a voluntary approach to smoke-free outdoor spaces, and creating more comprehensive by-laws over time. The time taken to allow the community and decision-makers to become familiar and comfortable with the issue and proposed solutions was important to ensuring that by-law development would move forward.

Being mindful of the current fiscal environment - Fiscal environments can either promote the need to address problems, pushing things higher up on the policy agenda, or constrain action and as a result keep things low on the agenda due to the perceived cost of addressing the problem (Kingdon, 2003). It was understood by policy entrepreneurs across the three cases that the perceived financial impact of a proposed by-law could impact its overall success. As a result, ways in which a by-law could be adopted with little cost to the municipality were highlighted drawing upon past successes with smoke-free space by-laws locally and in other municipalities. Decision makers and others gained a better understanding of how a by-law could be adopted with minimal financial repercussions, increasing willingness to move forward.

Rely on internal and external partnerships and collaborations - Municipal employees have many tasks and limited capacity to take on additional work related to outdoor smoke-free spaces. As employees of the municipality, there are also limitations placed on their ability to advocate for policy development. Collaborations and partnerships were used across the three cases to increase capacity to work on this issue, and to ensure that various perspectives were considered in the development process. In addition to increasing capacity to do the work, these collaborations also resulted in greater buy-in from key stakeholders which resulted in smoother implementation processes. Collaborations were formed with community advocacy groups and NGOs, other municipal committees and staff, and public health practitioners.

Frame issues so they are of interest to a wide variety of stakeholders – To ensure that many individuals could identify a reason to support the by-law, it was important that by-law development be framed to illustrate its relevance to many municipal departments, and not just public health. This was identified as critical as it may have made it easier for decision makers and others to identify the value of moving forward with by-law development. This was achieved by including messaging that focused on the environmental, economic, community development, and other impacts of outdoor tobacco use.

Highlight opportunities for innovation -The municipalities highlighted in this case study each had a strong history of previous smoke-free indoor space by-law work and expressed interest in continuing to build upon their previous tobacco control innovations and successes. The municipalities did this by adopting new policy restrictions that moved the gold standard for smoke-free outdoor space by-laws forward.

Mechanisms related to by-law implementation

Once municipalities had adopted a smoke-free outdoor space by-law, policy entrepreneurs and others remained engaged for the implementation of the by-law. A strong implementation process is essential to the success of the by-law and several mechanisms were found to be critical to implementation success:

***Create a shared vision and understanding among municipal employees* –** Many municipal employees work in locations where they are affected by smoke-free outdoor space by-laws. Employees may be required to respond to concerns or questions about the by-law, may need to deal with non-compliance, and if they themselves smoke, need to find a way to manage their smoking behaviours while on municipal property. Informing employees of the by-law and their role in its implementation, and providing opportunities for cessation support, were critical to ensuring the by-law was implemented smoothly.

Develop a strong communication strategy – In each of the cases studied limited funding was available to support proactive enforcement of by-laws. Based on past by-law experience, policy entrepreneurs understood that when community members are aware of a by-law, compliance increases. As a result, a strong emphasis was placed on increasing community awareness of the by-law which led to high compliance from the outset. This included a phased-in approach to enforcement beginning with an educational phase and the use of various communication strategies (paid and earned media, in-person visits, presence at community events) to increase awareness of the by-law.

Conclusions

Ten mechanisms were identified as important to the by-law development and implementation process across the cases examined. Policy entrepreneurs were critical to each of the mechanisms identified. They used these mechanisms to create and take advantage of policy windows, driving the by-law process forward. These policy entrepreneurs drew on scientific and practice evidence to demonstrate the need for smoke-free outdoor spaces. These processes were important to 'softening up' the community and decision-makers to pursue by-law development. They also highlighted their municipalities' history of successes around smoke-free indoor spaces which motivated the municipalities to be innovative and leaders in smoke-free outdoor spaces as well.

However, policy entrepreneurs did not work in isolation. Collaborations between diverse stakeholders allowed for increased capacity for by-law development work, and ensured the issue of smoke-free outdoor spaces was framed in such a way that many, including those outside of public health, would see it as an issue of interest. Framing the issue so it was clear that the by-law could be adopted with little fiscal impact on the municipality was also critical.

Strong communications were important to the success of the implementation phase. Communication with municipal staff to ensure they were aware of the by-law, and understood the role they played in its implementation was a strong focus. Further, communications with the general public were critical as it was understood that increased awareness would lead to increased compliance with the by-law, reducing the enforcement burden.

For smoke-free outdoor spaces to become the norm, additional municipalities must address outdoor tobacco use and pass comprehensive by-laws in this area. Public health practitioners and others within their communities can play an influential role. Identifying policy entrepreneurs that can drive the process and using the mechanisms that were important to the work completed by Barrie, Hamilton, and Ottawa can help to create a smoother path to by-law development.

Table of Contents

1.0 Introduction ... 1

1.1. Smoke-free space by-law development ... 1

2.0 Methods ... 3

2.1. Case selection ... 3

2.2. Data collection ... 4

2.2.1. Key informant interviews ... 4

2.2.2. Document review ... 4

2.3. Analysis ... 4

3.0 Results ... 5

3.1. The Actors - Policy Entrepreneurs ... 5

3.2. Mechanisms related to by-law development ... 6

3.2.1. Understanding the problem – drawing on evidence ... 7

3.2.2. Build on past successes: Moving from indoor to outdoor smoke-free spaces ... 9

3.2.3. Allow time for Council and the community to “soften up” on the issue ... 11

3.2.4. Be mindful of the current fiscal environment ... 14

3.2.5. Rely on internal and external partnerships and collaborations ... 15

3.2.6. Frame issues so they are of interest to a wide variety of stakeholders ... 16

3.2.7. Highlight opportunities for innovation ... 18

3.2.8. Create and take advantage of policy windows ... 19

3.3. Mechanisms related to by-law implementation ... 21

3.3.1. Create a shared vision and understanding among municipal employees ... 21

3.3.2. Develop a strong communication strategy ... 22

4.0 Conclusions ... 23

References ... 25

1.0 Introduction

Tobacco use remains a leading cause of preventable death and illness in Ontario. In 2002, an estimated 13,244 Ontarians died as a result of tobacco use. These deaths not only occurred among those individuals who used tobacco products; 315 of these individuals died as a result of lung cancer or ischemic heart disease caused as a result of exposure to second-hand smoke (SHS), and 36 infants under the age of one died as a result of conditions such as Sudden Infant Death Syndrome (SIDS) and conditions related to low birth weight or short gestation which arose as a result of exposure to tobacco in utero and/or SHS exposure (Rehm et al., 2006). Tobacco use not only negatively impacts the health of Ontarians, it is also extremely costly. In 2002, tobacco use cost Ontario nearly $6.1 billion dollars (Rehm et al., 2006). Given the broad impact that tobacco use has on the Ontario economy and the health of Ontarians, taking a population-level approach to reduce the use of tobacco is warranted. Policy changes are one way that tobacco use can be addressed at the population level. There is a wealth of evidence supporting the use of smoking restrictions in public places (Mozaffarian et al., 2012). Ontario has a strong history of using policy to protect individuals from exposure to SHS, to encourage those who smoke to quit, and to prevent those who do not smoke from starting to do so. Currently in Ontario many municipalities and regions are developing new by-laws to further protect individuals from exposure to tobacco, specifically in outdoor spaces. This paper presents the results of a multiple case study examining the mechanisms associated with the development of three comprehensive smoke-free outdoor space by-laws in Ontario.

1.1. Smoke-free space by-law development

Smoke-free space policies have been adopted at all levels of government within Ontario. In 1994, *the Tobacco Control Act* (TCA) was passed and this amended the *Municipal Act* explicitly giving municipalities the authority to adopt by-laws creating smoke-free workplaces and public places (OCAT, 2012). As a result, municipalities and regions began to adopt by-laws addressing tobacco use. This initial period of by-law development concentrated on the creation of smoke-free indoor places and workplaces. The wave of by-law development at the local level in Ontario provided those in higher levels of government with evidence demonstrating that they should move forward with legislation at the provincial level (Nykiforuk et al., 2010). Province-wide restrictions on smoking in indoor places were adopted as a result of the *Smoke-Free Ontario Act* (SFOA) which came into effect in May 2006. The SFOA prohibits smoking in indoor places and workplaces, on outdoor patios with a complete or partial roof, on school grounds, within 9 metres of entranceways to hospitals, health care and psychiatric facilities, in public areas of multi-unit dwellings and in private home daycares. As of January 21, 2009, smoking is also prohibited in cars when individuals under the age of 16 years are present (Ministry of Health and Long-Term Care, Government of Ontario, 2011). Prior to the implementation of the SFOA, approximately 9 out of 10 Ontarians lived in a municipality or region that had adopted their own local by-law creating smoke-free restaurants and/or bars. The SFOA increased these protections in many cases as it did not allow for designated smoking rooms (DSRs) while just over half of local level by-laws permitted DSRs (Ontario Tobacco Research Unit [OTRU], 2012). Indoor smoke-free policies have been associated with positive changes at the population health level including reduced rates of hospital admissions for childhood asthma (MacKay et al., 2010) and for heart disease (Lippert & Gustat, 2012), reduced risk of acute myocardial infarction (Lippert & Gustat, 2012; Meyers, Neuberger & He, 2009), and reduced rates of smoking (Lippert & Gustat, 2012; Wilson et al., 2012).

Although restrictions on smoking indoors have reduced Ontarians' exposure to SHS, exposure remains an issue. In 2012, 32% of Ontarians reported exposure to SHS on restaurant patios, 53% reported exposure at building entrances and 57% reported exposure to SHS outdoors (e.g., on a sidewalk or at a park) in the past month (OTRU, 2012).

Ontarians appear to be ready for additional regulations on where smoking is permitted. According to a 2011 Ipsos Reid poll, 89% of Ontarian respondents indicated that they would support additional tobacco control measures such as prohibiting smoking in areas where children play (Ipsos Reid, 2011).

When individuals smoke outdoors, levels of SHS can be high. Given that there is no safe level of exposure to SHS (USDHHS, 2006), this is concerning for the health of the population. A study by Klepeis, Ott & Switzer (2007) found that measurements of SHS outdoors could be quite high when in close proximity to a lit cigarette. When standing near a lit cigarette, levels of SHS measured were comparable to indoor concentrations, although the levels dropped quickly once the cigarette was extinguished. Several studies have measured levels of SHS outdoors in areas including the entrances to buildings (Kaufman et al., 2011) and restaurant patios (Cameron et al., 2010; Kennedy, 2010). Studies demonstrated that when cigarettes were smoked outdoors, average levels of $PM_{2.5}$[1] were significantly higher than existing background air pollution levels in the areas around the lit cigarette (Cameron et al., 2010; Kaufman et al., 2011; Kennedy, 2010) and that the measured levels increased as the number of lit cigarettes in the area increased (Cameron et al., 2010; Kaufman et al., 2011).

Outdoor smoking restrictions not only reduce exposure to SHS, they may also decrease the risk of fire, reduce tobacco product related litter, and protect community members from nuisances (Bloch & Shopland, 2000). It is also noted that smoking bans in outdoor spaces can reduce social exposure to tobacco use. One study explored where youth and adults witnessed smoking and the perceived social acceptability of smoking. The authors found that the more youth witness smoking, the more they will consider it to be socially acceptable suggesting that "*smoking bans have the potential to change established social norms*" (Alesci, Forester & Blaine, 2003).

The Smoke-Free Ontario-Scientific Advisory Committee's (SFO-SAC) Report released in 2010, which provides recommendations to inform the renewal of the provincial tobacco control strategy for 2010 to 2015 also recommends the amendment of the SFOA to eliminate smoking of tobacco products and the use of waterpipes in restaurant and bar patios and selected outdoor places. Selected outdoor places include doorways to commercial and public buildings, transit shelters, parks and playgrounds, outdoor sport facilities, beaches, sidewalks, outdoor entertainment venues and public events (SFO-SAC, 2010).

As a result of this knowledge, a second phase of by-law development is occurring in Ontario. This phase of by-law development focuses on the creation of smoke-free outdoor spaces including smoke-free parks, playgrounds, municipal properties, beaches and restaurant patios. As was the case when smoke-free indoor spaces became the norm, municipalities and regions across Ontario are taking the lead to create smoke-free outdoor spaces. As of the fall of 2012, over 75 municipalities and regions in Ontario had implemented by-laws or policies related to tobacco that extended beyond the SFOA, with the majority addressing smoking in outdoor places (Non-Smokers' Rights Association (NSRA), 2012). As a result, over 65% of Ontarians are protected from exposure to SHS beyond the protections afforded by the SFOA. The level of additional protection, however, varies significantly between municipalities with some restricting smoking to a certain distance from municipal buildings, and others creating 100% smoke-free parks, playgrounds, sport fields and restaurant patios. Little is known about how these by-laws are implemented, and what mechanisms are associated with the development of comprehensive by-laws. This documentation of practice project sought to gain additional insights into these issues. Using a multiple-case design, the project sought to address the

[1] $PM_{2.5}$ is particulate matter that measures less than 2.5mm in diameter and is often used as a proxy measure for second-hand smoke. This is produced by cigarettes when they burn. It is not specific to cigarettes, and is also created by other sources. As a result, it is important to consider what the background level is, and how this changes when cigarettes are smoked to understand the contribution of tobacco to the $PM_{2.5}$ measurements.

following research question: **What contexts and mechanisms are associated with the development of by-laws with high levels of regulatory strength?**

2.0 Methods

This study aimed to understand the mechanisms associated with the development of smoke-free outdoor space by-laws. Taking a realist approach (Maxwell, 2004; Pawson & Tilley, 1997), the study aimed to understand what works, for whom and under what conditions to develop smoke-free outdoor space by-laws. A holistic, multiple-case design (Stake, 2006; Yin, 2009) was used to investigate this topic.

2.1. Case selection

Given that the phenomena of interest for this study was the mechanisms associated with the development of smoke-free outdoor space by-laws, three examples of smoke-free outdoor space by-laws were selected for inclusion. The Non-Smokers' Rights Association (NSRA) has developed a database of by-laws at the municipal and regional levels that go beyond the requirements of the SFOA to create smoke-free spaces and served as the initial sampling frame to identify cases for inclusion in the study[2].

To be eligible for inclusion in the study, municipalities were required to have taken actions to create smoke-free outdoor spaces, and the restriction needed to be implemented through a by-law rather than a policy. By-laws that were eligible for inclusion were ranked by regulatory strength. Regulatory strength was determined based on the number of places where smoking was restricted, and the strength of those restrictions (complete ban, partial ban). Final cases were selected from the by-laws with the highest levels of regulatory strength. Six municipalities/health units were approached regarding participation, and three agreed to participate. The three cases included the municipalities of Ottawa, Hamilton and Barrie. Table 1 outlines the characteristics of each case. In Ottawa a series of by-law amendments were passed to create smoke-free outdoor spaces.

Table 1. Key Characteristics of cases

Characteristic	Barrie	Hamilton	Ottawa
Population[a]	135,711	519,949	883,391
Smoking rate (2011, by LPHA)[b]	21%	19.5%	15%
By-law passed	May 2009	March 2011	February 2012
By-law implemented	July 2009	May 2012	April 2012
Smoke-free areas	Municipal property including parks, playing fields, beaches, and municipal building properties, DSAs in selected parking lots	Municipally owned park and rec properties including playgrounds, rec centres, arenas, playing fields, stadiums, pools, dog parks	Bar and restaurant patios, municipal property including parks, beaches, events, outdoor markets, transit property (as of 2007)
Types of tobacco products included	Pipes, cigars, cigarettes (Excludes smoking for theatrical purposes)	Pipes, cigars, cigarettes, cigarillos, use of hookah pipes	Pipes, cigars, cigarettes<br>Note: A separate by-law prohibits hookah use on municipal property

[a] Statistics Canada, 2013

[b] Ontario Tobacco Research Unit, 2012

[2] The database can be found at: http://www.nsra-adnf.ca/cms/smoke-free-laws-database.html

Each municipality's by-law can be seen as being innovative. Barrie was among the first municipalities to adopt a comprehensive by-law that went beyond smoke-free buffer zones around buildings or playgrounds to create broader smoke-free spaces. They were also among the first to include beaches in the definition of parks or municipal property. Hamilton was the first municipality to explicitly include hookah/waterpipes in their by-law when defining tobacco use. Ottawa has the most comprehensive smoke-free outdoor space policy in Ontario. They are one of the few municipalities to include patios on the list of smoke-free outdoor spaces.

2.2. Data collection

2.2.1. Key informant interviews

Telephone, audio-recorded semi-structured interviews that lasted 30 to 90 minutes were conducted with two to six key informants per case (total of ten interviews with eleven key informants across the three cases) who were involved in the by-law development, adoption and implementation processes. Multiple perspectives about the by-law process were captured to discern whether emerging mechanisms were consistent across the key informants, which served as the primary data source. Key informants included municipal staff, community advocates and public health practitioners. Initial contact was made with individuals from the health unit and/or community who were known to be involved in the by-law development process and they referred additional key informants who were contacted to determine interest in participation.

2.2.2. Document review

At the time of recruitment, key informants were asked to share documents with the project team regarding the development, adoption and implementation of the by-law. Documents were used as a secondary source of data to triangulate the information provided in the interviews. Examples of documents provided included: copies of the by-law, supporting materials, work plans, reports to council, and promotion plans. A subset of these documents, specifically communication tools that others may be able to adapt and utilize in their own context, are included in Appendices A - C. Written informed consent was obtained prior to interviews from all individuals participating in the study and for inclusion of the documents collected in this report.

2.3. Analysis

The qualitative analysis software NVivo 9.2 was used to guide the analysis process. Two levels of analysis were conducted. The first level involved an analysis of the data at the case level to allow for the identification of the key mechanisms associated with the development of the smoke-free outdoor space by-law within each of the three municipalities' specific contexts. The Health Communication Unit's (THCU) model of policy development was used to structure the analysis. The analysis also allowed for other themes to emerge from the data. During this process, transcripts from the key informant interviews and the documents provided by informants both served as key sources of data. The results of this analysis are presented in the three case descriptions found in Appendices A – C. These case descriptions were reviewed and approved by the key informants and may serve as models for replication in other jurisdictions that share similar contexts and circumstances.

The second level of analysis was the cross-case analysis. This level of analysis sought to understand the key mechanisms that played a role in the development of the smoke-free outdoor space by-laws across the three contexts. Understanding what mechanisms were critical to the development of the outdoor by-law regardless of context can provide broader insights that individuals in the field can use to understand the development of such by-laws and potentially incorporate into their approaches.

3.0 Results

The following section outlines the key mechanisms that were found to be associated with the development of smoke-free outdoor space by-laws across the three cases. Variations in the ways in which the mechanisms played out within the cases are highlighted. Overall, the policy development process across the cases fits with the model of policy development created by Kingdon (2003). Kingdon suggests that within politics there are three main streams – the problem, policy and political streams. These streams are typically largely independent, but when each of the three streams converge, namely *"a problem is recognized, a solution is available, the political climate makes the time right for change, and the constraints do not prohibit action"* (Kingdon, 2003, p. 88) a policy window is opened and there is an opportunity for policy making.

In each of the three cases examined for this study, a policy window opened and this was capitalized on to move the smoke-free outdoor space agenda forward. The mechanisms highlighted in this paper each played a role in moving the issues, policy options and political agenda forward to allow for the three streams of policy development to converge and create a policy window. Elements of each of the mechanisms fired throughout the by-law development and implementation phases. Many of these mechanisms, however, did apply more strongly to either the by-law development or by-law implementation phases. As a result, the mechanisms are presented based on the stage of by-law development to which they applied most strongly.

3.1. The Actors - Policy Entrepreneurs

According to Kingdon (2003), policy entrepreneurs are individuals with a *"willingness to invest their resources – time, energy, reputation and sometimes money - in the hope of a future return"* (p. 122). Policy entrepreneurs are part of the '*community of specialists'* who take on champion roles and help bring forward policy options. The importance of champions in the development and implementation of the smoke-free outdoor space by-laws cannot be minimized. Policy entrepreneurs were critical throughout both the by-law development and by-law implementation phases.

Policy entrepreneurs across the three cases fell into the following categories: public health practitioners, City Councillors, community advocates, and youth. These individuals pushed the agenda forward, provided support and information to decision makers and ensured the public was aware of the need for smoke-free outdoor spaces. Across the three cases, it is clear that without policy entrepreneurs, policy development may not have occurred. Moreover, different categories of entrepreneurs initiated the policy development process in the different cases and as such, any entrepreneur can be the driver of initial change. The different categories of entrepreneurs and the roles they played in each case are outlined below.

Public health practitioners: Public health practitioners played the role of policy entrepreneurs for smoke-free outdoor spaces across all three cases. Working within the government structure, they encouraged the development of smoke-free spaces. They raised awareness among community members, consulted the evidence and built the support needed to ensure that smoke-free outdoor spaces would become a reality through by-law development. Public health practitioners were critical to the by-law development process. As noted by a key informant, *"in terms of pushing the policy agenda, Public Health was, you know, the leader in that…everyone else came onboard but wouldn't have pushed the agenda without Public Health"* (Hamilton).

City Councillors: In all three cases, City Councillors played a champion role. In Ottawa, one City Councillor drew Council's attention to the issue of smoke-free outdoor spaces by asking the municipality to consider creating smoke-free outdoor spaces and smoke-free patios after 8:00 pm each night. This motion paved the way for Public

Health to examine the issue and for the Board of Health to recommend a by-law. In Barrie, it was City Council who started the ball rolling for the issue of smoke-free outdoor spaces to be examined, and they were the individuals who pushed for a more comprehensive by-law to be developed. In Hamilton, City Council identified a desire to be innovative in the area of tobacco control and asked Public Health Services for their insights into the best areas of action for the city in regards to tobacco use. As noted by a key informant, *"the time was right [for by-law development] and the councillors who were champions kind of seized it and moved forward"* (Barrie). Having Councillors as champions was especially beneficial given that they are the ones who had to make the final decisions regarding by-law development.

Community advocates: Ottawa is a unique case in the sense that they have a strong history of community advocacy in the area of tobacco control. Ottawa's position as the capital of Canada ensures rich access to a variety of different advocacy groups, non-governmental organizations and political leaders. As noted by one key informant, Ottawa is *"one of the few areas in Ontario that has a local Council on Smoking or Health and we have the advantage that we have some national organizations and folks that also live in this community…important people with strong advocacy skills and knowledge of tobacco control"* (Ottawa Public Health [OPH] Staff). As a result, in the Ottawa case community-based advocates played a strong role in the by-law development process and became strong policy entrepreneurs, something that was not seen in the two other municipalities studied. The Ottawa Council on Smoking or Health (OCSH) has played a major role in by-law development in the past and continued this work advocating for smoke-free outdoor spaces. As policy entrepreneurs, OCSH members helped raise the profile of tobacco use in outdoor spaces to encourage City Councillors to pay attention to the issue as a problem of interest, they engaged the community to advocate for smoke-free outdoor spaces, and they ensured the issue remained on the policy making agenda. It was noted that as community members, OCSH members were in a better position to advocate for smoke-free outdoor spaces, compared with municipal employees who are limited in the advocacy activities they can undertake.

Youth: Barrie was the only case where youth were identified as being key policy entrepreneurs. In Barrie, the Youth Action Alliance (YAA) identified the issue of smoke-free outdoor spaces as an issue of interest and completed activities to raise awareness of the issue. The youth involved with the YAA identified their own priority area of work and focused on tobacco use in outdoor spaces as *"they felt that it was something that had a negative impact....so they felt very passionately about helping Barrie develop a by-law"* (SMDHU Staff). Youth spread awareness of the need for smoke-free outdoor spaces through personal conversations with individual community members and through media activities, and demonstrated community support for by-law development through a paper and online petition.

Recommendations for future action:

- Identify "policy entrepreneurs" (champions) who are willing to invest resources in the campaign for smoke-free spaces; ideally at least one policy entrepreneur should be identified from both City Council and public health.
- If the municipality has a strong advocacy community encourage those individuals to get involved in the push for smoke-free outdoor spaces. Involving youth in the process may also be beneficial.

3.2. Mechanisms related to by-law development

The following mechanisms were highlighted as playing a critical role in the by-law development process – from the time when the need for a smoke-free outdoor space by-law was identified through to the adoption of the smoke-free

outdoor space by-law. As noted earlier, aspects of these mechanisms may also apply to other phases of the by-law process, such as by-law implementation, and thus should be considered throughout the by-law processes.

3.2.1. Understanding the problem – drawing on evidence

Context:

There was a sense that communities had a strong understanding of the dangers of SHS exposure, especially indoors, but less understanding of the impact of outdoor exposure to SHS. There were concerns about the feasibility of an outdoor by-law.

+

Mechanisms:

Literature reviews, primary research, consultations with scientific experts were used to deliver scientific evidence on smoke-free outdoor spaces to the community and decision makers.

Environmental scans of and consultations were conducted with other jurisdictions to learn about their smoke-free outdoor space by-laws and experiences with by-law development, implementation and enforcement

→

Outcomes:

Outdoor SHS exposure identified as a topic of concern broadly and at the local level; this led to an understanding of others' success in this area, and how to move forward with by-law development locally.

According to Kingdon (2003), for a condition to be viewed as a problem it is important that decision makers understand that this is an issue that can and should be addressed. One way of doing this is to highlight evidence suggesting that there is a problem that can be rectified. Key informants from all three cases studied highlighted the value of both practice and scientific evidence throughout their narratives for identifying a problem, and for suggesting that the problem could realistically be addressed. Neither the scientific nor practice-based evidence were used alone; in all three cases, both types of evidence were highlighted as they played different and complementary roles in the process. Practice-based evidence enabled other municipalities to observe others successful approaches and the ease with which they were developed, implemented and enforced. Scientific-evidence enabled municipalities to understand that tobacco use in outdoor spaces was an issue of concern, and that there was evidence to support action in this area.

Municipalities seemed to be more receptive to the idea of smoke-free outdoor spaces and the information that was gleaned from the scientific and practice evidence because the ground work establishing SHS as a danger had already been laid out during efforts to create smoke-free indoor spaces. It was noted by key informants that there was a strong understanding within the community and among decision makers that exposure to SHS was dangerous. As a result of this, it was easier to get the public and decision makers to understand how the dangers of SHS and tobacco use translated to the context of tobacco use in outdoor spaces. As one key informant noted, *"I think the acceptance that second-hand smoke is a hazard, a danger…I think the acceptance is just much higher. I don't think anyone's going to argue that they want to be exposing children to second-hand smoke"* (Ottawa).

Scientific evidence

Scientific evidence, in many cases, spurred on by-law development. In two of the three cases highlighted, key informants spoke of how there was *"more research coming out about the effects of second-hand smoke outdoors"*

(Hamilton) and how this provided rationale to move forward with by-law development. The importance of understanding the scientific evidence related to exposure to SHS in outdoor spaces was emphasized. Each municipality conducted literature reviews to understand the scientific evidence. In one case, primary research was conducted to demonstrate how smoking on a restaurant patio impacted levels of exposure to SHS and reinforce the value of and need for smoke-free patios (Ottawa; See Appendix C for more information). Scientific evidence was shared with decision makers and the broader community in a variety of ways including:

- Letters from the Medical Officer of Health (Barrie) and from advocacy groups (Ottawa) to City Counsellors and the Mayor
- Reports to the Board of Health/City Council (Barrie, Hamilton, Ottawa)
- Deputations to Council and the Board of Health by researchers and other experts (Barrie, Hamilton, Ottawa)
- Media releases and earned media on the primary research (Ottawa)
- Youth-driven promotional events (Barrie)

Practice-based evidence

Practice-based evidence, that is the knowledge gained from others who have adopted smoke-free outdoor spaces, was also critical. A preliminary step in the by-law development process in all three cases was an environmental scan to understand what actions other municipalities were taking in regards to smoke-free outdoor spaces. This knowledge was used to build the case for by-law development, implementation and adoption. Kingdon (2003) notes that one strategy for moving a condition to become a problem can be the use of comparisons – comparing the current jurisdiction to another who has already addressed the problem of interest. In all three cases, key informants noted in their narrative that the work done by other municipalities may have influenced the way that their municipality took on the issue. For example, in Barrie it was noted that decision makers were able to see how another local municipality (Collingwood) had adopted smoke-free outdoor spaces successfully, and this encouraged them to also take action. As a key informant noted, *"Collingwood was a huge factor, they are not too far away from us and they had such positive reviews and they had such a good process*" (Barrie).

Practice-based evidence was further used to provide support to the arguments that the smoke-free outdoor space by-law could be easily developed and adopted, that enforcement was not a major issue, and that the by-law could be successful. In Ottawa, public health practitioners and other municipal staff held teleconferences with staff from Vancouver and New York, two cities which had adopted smoke-free outdoor space by-laws, to understand the implementation process and the challenges and successes they had faced. This information was then used to confirm that smoke-free outdoor spaces were feasible, and could be implemented. As a key informant noted, this information helped them to realize, *"if these larger cities could pass a by-law, certainly the City of Ottawa could also do so and protect the health of their residents"* (Ottawa).

Both practice and scientific evidence played a role in defining the problem and ensuring it was seen as a relevant and important problem to decision makers, identifying possible policy solutions to the issue, understanding how these solutions could work in practice, and understanding the social norms surrounding outdoor tobacco use within the province and internationally.

Recommendations for future action:

- Highlight both scientific and practice-based evidence to make the case for smoke-free outdoor spaces.
- Consult with other municipalities who have developed smoke-free outdoor space by-laws and showcase their successes and the ease with which this can be successfully executed, including experiences with the development, implementation, enforcement and any budgetary concerns.

3.2.2. Build on past successes: Moving from indoor to outdoor smoke-free spaces

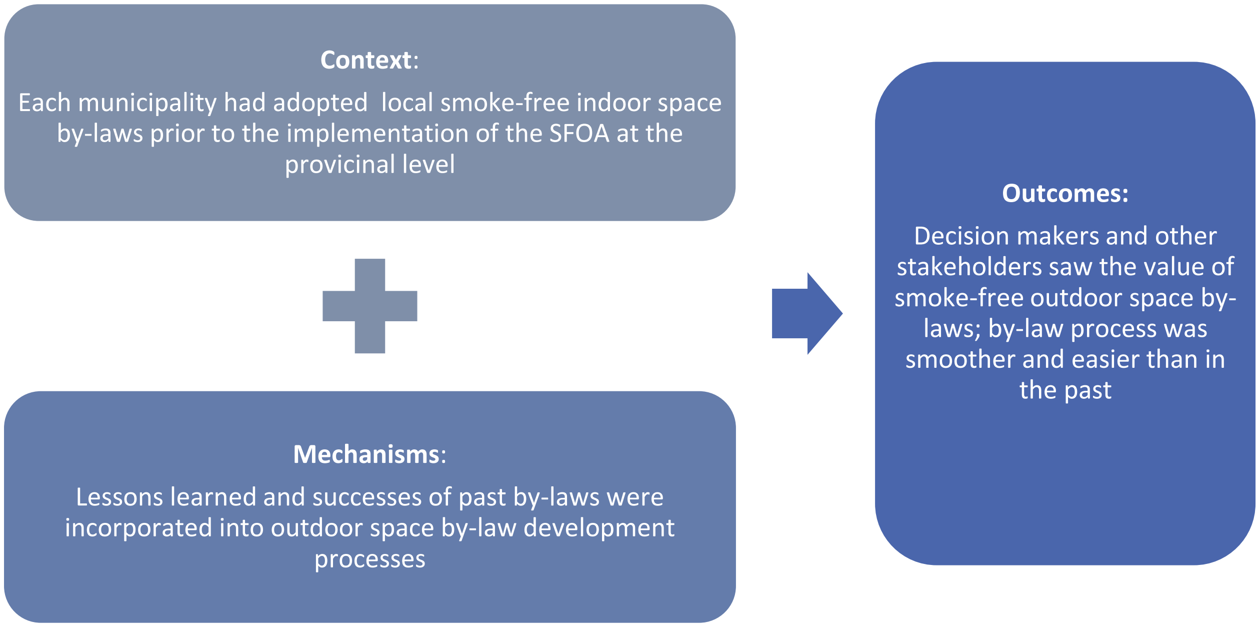

As noted in the introduction, the development of smoke-free outdoor space by-laws is part of the second wave of by-law development by municipalities in the area of smoke-free spaces. The first wave of by-law development resulted in the creation of smoke-free indoor spaces. The municipalities studied played an active role in the first wave of by-law development, each adopting their own smoke-free indoor space by-law prior to the implementation of the SFOA at the provincial level in 2006. Ottawa's by-law was implemented August 1, 2001 and was the first 'gold' standard by-law to be passed in Ontario. All restaurants, bars, bowling alleys, billiard halls and bingo halls were 100% smoke-free with no designated smoking rooms (DSRs) permitted. Hamilton and Barrie passed smoke-free indoor space by-laws on June 1, 2002 and June 1, 2003 respectively. Both cities initially implemented 'silver' level by-laws which allowed for exemptions for specific types of establishments and the inclusion of DSRs. Both cities' by-laws included dates when they would move to 'gold' standard level with no DSRs and exemptions permitted, but the SFOA, which prohibited DSRs, was passed prior to either of the city's 'gold' implementation dates being reached (OCAT, 2013).

Key informants from all three municipalities highlighted the value that this previous by-law work brought to the efforts to develop smoke-free outdoor spaces. Working in a context where smoke-free space by-laws had already been implemented was seen as making the overall process easier, more straight forward and less costly. Highlighting the lessons learned from this past work was identified as being incredibly beneficial.

Kingdon (2003) highlights the fact that when a success is achieved in one area, it often sets a principle that affects future policy development. The adoption of the smoke-free indoor space by-laws have set a principle that the public and decision makers are able and willing to regulate where tobacco products can be used. The principle made the

second wave of by-law development easier because the indoor smoke-free spaces campaign successfully shifted social norms around the issue. The public and decision makers were already educated about and accepted that second-hand smoke exposure had a negative impact on citizen's health and that smoke-free spaces serve as one approach to consider when aiming to improve health outcomes. One key informant noted, *"I think the success we had with the smoke-free workplaces and public places by-laws pre-SFOA – that was like walking up Kilimanjaro. In comparison, this was like going to the hills in Collingwood. I like to think that's because of the amount of work we put into the 2003 [smoke-free indoor space] by-law"* (Barrie).

Past by-law work was also identified as an influence on the scope of the by-law in two of the three cases. When developing smoke-free outdoor space by-laws there is the opportunity to recommend a by-law with a narrow scope (e.g., buffer zones around municipal buildings) or to recommend a broad by-law which creates many smoke-free spaces. Key informants from two cases spoke of how they used their past experiences with smoke-free space by-laws to recommend the development of a broad by-law. One case created buffer zones around particular priority areas, rather than adopting a 100% smoke-free by-law. Public health professionals from this case, however, noted that when the smoke-free indoor space by-laws were adopted a narrower by-law was created which allowed for DSRs and a few years later as the evidence grew on the need for 100% smoke-free indoor spaces, the by-law needed to be changed. To avoid having to go through that process again, they opted for a more comprehensive by-law from the outset. It was also noted that having a by-law that is 100% smoke-free is easier to communicate to the public than a by-law that creates some smoke-free areas. Key informants in a second case spoke passionately about how their municipality had been focused on moving towards smoke-free outdoor spaces since the initial implementation of the smoke-free indoor space by-law. The history of adopting an innovative by-law to create smoke-free indoor spaces was linked with the desire to also push for the 'gold standard' in an outdoor space by-law.

The importance of the SFOA and municipal smoke-free indoor space by-law's legacies in making the smoke-free outdoor space by-law process move more smoothly was highlighted by those who are strongly rooted in the tobacco control field (public health practitioners, advocates). Those working in other municipal departments did not highlight this history as being important contextually, nor as a mechanism to move by-law development forward.

Recommendations for future action:

- Emphasize the success that was obtained from the smoke-free indoor space by-laws – highlight the ease of enforcement, the minimal negative side effects, changes in public perception, smoking rates
- Learn from the past work – understand which decision makers may view additional smoke-free space regulations favourably, prepare for opposition from individuals who opposed past by-laws, consider lessons learned and successes from the past when determining the scope of the by-law and how it will be implemented.
- Emphasize to decision makers that taking action in the area of smoke-free indoor spaces prior to the SFOA may have allowed them to be seen as progressive and as municipalities that took on leadership roles in tobacco control. Moving forward with smoke-free outdoor spaces may allow them to continue to maintain that image, by demonstrating that they continue to be innovative and progressive municipalities focused on tobacco control and the health of their citizens.

3.2.3. Allow time for Council and the community to "soften up" on the issue

Context:

Although the need for smoke-free outdoor spaces was identified, the by-law development process was lengthy and in some cases political will needed to be developed.

City Councillors needed to be responsive to the "mood" of the community when making decisions on priorities

+

Mechanisms:

Information about smoke-free outdoor spaces was shared with the community and decision makers.

The need for smoke-free outdoor spaces was highlighted by demonstrating what other municipalities were doing and the changing social norms related to tobacco use outdoors; voluntary policy development was a focus until the by-law was in place. These actions were highlighted to decision-makers to show a readiness for by-law.

City Councillors were made aware of community support for by-law development through various tactics

→

Outcomes:

Decision makers and the community were aware of the need for smoke-free spaces, and were ready to move forward with comprehensive by-laws when the time was right.

Decision makers were aware of the community mood which made the decisions regarding how to move forward more straight forward

Kingdon (2003) asserts there is a strong advantage to ensuring that decision makers and the public become aware of the proposed solution to the problem so when a policy window does open, there is readiness for by-law development. This is referred to as the "softening up" process. In all three cases, actions were taken to begin to "soften up" decision makers and the public in regards to smoke-free outdoor spaces. Activities undertaken to allow for this "softening up" process included:

- ***Changing social norms related to tobacco use and continuously highlighting the need to further protect citizens by expanding tobacco control policies including smoke-free spaces***: Each municipality examined in this study had adopted smoke-free indoor spaces in the past, which involved a strong emphasis on educating the public and decision makers of the importance of smoke-free spaces and the dangers of SHS exposure. Key informants noted that through this work the social norms related to tobacco use changed and the community generally had a stronger understanding of the issues surrounding tobacco use and the importance and value of smoke-free spaces. Changes in social norms was one way in which the community and decision makers could be "softened up" as the understanding created from past work made it easier for groups to accept the need for smoke-free outdoor spaces.
- ***Beginning with a voluntary approach to smoke-free outdoor spaces***: While waiting for the political will to move forward with by-law development, a focus was placed on voluntary smoke-free policies. In Ottawa, OPH staff focused on the development of voluntary smoke-free outdoor space policies and on educating the public on the issue of tobacco use outdoors. As a result, prior to adoption of the smoke-free outdoor space by-law amendments, 284 parks in Ottawa were voluntarily smoke-free as were numerous restaurant patios. The

individuals involved in this voluntary work became advocates who could share the benefits of smoke-free outdoor spaces during the by-law development phase. In Hamilton, various sport teams also created their own tobacco-free policies stating that they would not smoke outdoors while participating in sport and recreation activities. Across the province, the Tobacco-Free Sport and Recreation (TFSR) movement was occurring which encouraged the development of voluntary tobacco-free policies for sport and recreation organizations. Various tobacco control area networks (TCANs) and health units across the province were engaged in this work. This contributed to the development of voluntary smoke-free parks in the Ottawa case.

- ***Educating decision makers:*** Advocates in Ottawa played a major role in the softening up phase. When the OCSH determined that they wanted to begin to push for smoke-free outdoor spaces, decision makers were not yet ready to take that step. OCSH spent a year sending information to City Council Members, publicizing the need for smoke-free outdoor spaces and collecting information on the community readiness for this type of policy. In Barrie, it was noted that discussions about the next step for smoke-free outdoor spaces were discussed at the Board of Health meetings which some Barrie City Councillors attend. In Hamilton, public health practitioners shared information about smoke-free outdoor spaces with council when asked to identify additional areas of work for tobacco control in the city.
- ***Beginning with a less comprehensive approach to by-law development, and moving to the comprehensive level:*** In all three cases, the original request from Council to the municipal staff asking for the consideration of a smoke-free outdoor space by-law was less comprehensive than the by-law that was ultimately passed. In Ottawa the original motion from council asked for public health to report back on the development of a by-law that would apply prior to 8:00pm, in Hamilton and Barrie the original reports were to consider the adoption of buffer zones around specific areas. Through the by-law development process, however, Council agreed to expand the scope of the by-law to be more comprehensive. The mechanisms highlighted above (educating decision makers, changing social norms and beginning with a voluntary approach) may have contributed to decision makers' willingness to move forward with a more comprehensive by-law. Initially considering the less comprehensive by-law may have also served as an opportunity for decision makers to open the conversation and to gauge the openness of the community to such a by-law increasing the comfort with and "softening" them up to the idea of moving forward with a more comprehensive approach.

The activities highlighted under this mechanism all relate to how a community, including decision makers, can work to "soften up" an issue before it is brought forward for a final decision. Spending the time building on the successes of the first wave of by-law work around smoke free spaces, laying the ground work for the second wave of by-law work through education, voluntary approaches, consideration of various policy options and overall opening the dialogue on smoke-free spaces may make the process of creating a smoke-free outdoor space by-law smoother and easier.

Demonstrating community readiness for by-law development

The mechanisms associated with softening up all contribute to ensuring that decision makers are aware of the community's readiness to move forward with smoke-free outdoor spaces. Across all three cases, it was critical to ensure that decision makers had evidence of community members' readiness to move forward. As one key informant noted, *"you cannot get a by-law passed without the Counselors' support. And most of them will support you if they know their constituents are behind them. So, that is the key thing, the key thing in the campaign is to have the public make a statement that they want change"*. Kingdon (2003) emphasizes the importance of decision makers being aware of the 'national mood' and its role in moving a problem to the front of the policy making agenda. He describes the national mood as *"a large number of people…thinking along common lines"* and notes that this mood is continuously changing, and "*these changes in mood or climate have important impacts on policy agendas and policy*

outcomes" (Kingdon, 2003). Local evidence and consultations were used to inform decision makers of the 'mood' of the community elevating the issue to one that should be considered a problem and addressed.

The mechanisms that were used to 'soften up' the community and decision-makers also played a role in demonstrating community readiness. Additional mechanisms were also undertaken including:

- Acknowledging that the municipality and/or health unit were receiving a number of complaints related to tobacco use in outdoor spaces (Ottawa & Hamilton)
- Encouraging community members to contact the City Councillors by phone or e-mail to express their support for smoke-free outdoor spaces (Ottawa & Barrie)
- Formal consultations with the community members to understand the level of interest in smoke-free outdoor spaces (Ottawa & Hamilton)
- Opinion polls to allow for local level statistics on the views of the community, on the need for smoke-free outdoor spaces (Ottawa & Hamilton)
- Petitions and/or postcards signed by community members to be shared with the Mayor and/or City Council to demonstrate support for smoke-free outdoor space by-law development (Barrie & Ottawa)
- Delegations by individual community members at Council meetings when decisions regarding smoke-free outdoor spaces are being made (Ottawa, Hamilton, Barrie)
- Youth events to draw community and Council attention to the issue of smoke-free outdoor spaces (Barrie)

Recommendations for future action:

- Before moving into by-law development, spend time making the public and decision-makers aware of the need for smoke-free spaces. It is critically important to demonstrate to decision makers that the community is ready to address the problem of interest.
- Consider taking a voluntary approach to smoke-free spaces (e.g., smoke-free community parks, smoke-free teams) to build momentum for smoke-free spaces until there is political will to move forward with by-law development.
- Find ways to engage community members in the by-law development process to ensure that City Counsellors are aware of their interest in smoke-free outdoor spaces. Community members can be actively engaged in the process by participating in consultations, writing e-mails/letters to their Counsellors or advocating for smoke-free outdoor spaces, or they can be more passively engaged in the process (participating in a survey for example).

3.2.4. Be mindful of the current fiscal environment

Context:

Municipalities were limited in what issues and problems they could address as a result of their fiscal responsibilities. It was understood that the perceived financial impact of a proposed by-law may impact its overall success.

Mechanisms:

Ways in which a smoke-free outdoor space by-law can be adopted with little cost to the municipality were highlighted with emphasis being placed on past successes with smoke-free space by-laws locally and in other municipalities.

Outcomes:

Decision makers and others had a better understanding of how a by-law could be adopted with minimal financial repercussions which may have increased the willingness to move forward.

Budgets can act as a promoter of problems, pushing things higher up on the policy agenda but it can also act as a constraint, keeping things low on the agenda due to the perceived cost of addressing the problem (Kingdon, 2003). In all three cases highlighted, decision makers did not question the appropriateness of the by-law, but many raised concerns around the fiscal impact of such a by-law. Some key informants noted the fiscal environment played a significant role in this phase of by-law development compared with the role it played in the development of smoke-free indoor spaces. This context impacted the way in which the by-law was pitched to decision makers and the way it was implemented.

To ensure fiscal issues did not act as a constraint to by-law development, key informants highlighted various strategies they utilized to convey that the proposed by-law posed minimal fiscal impacts for the municipality. These strategies included:

- ***Emphasizing the self-enforcing nature of the by-law***: A key fiscal concern centred on enforcement. It was unclear to many decision makers how a by-law which would impact so many areas would be enforced without a large fiscal investment. Key informants from all three cases spoke of how they emphasized the self-enforcing nature of the proposed by-law to decision makers to reduce these concerns. The experiences of other municipalities who had adopted similar by-laws suggested that the by-law would not require a significant amount of resources or proactive enforcement, but rather would be self-enforcing with citizens generally complying with little proactive enforcement. Additionally, the municipalities experiences with adopting smoke-free indoor space regulations in the past (municipal by-laws and the SFOA) found that these by-laws were also largely self-enforcing. Key informants noted that they communicated this information to decision makers to reassure them that the by-law was enforceable with a limited budget. To make this explicit to decision makers, by-law enforcement staff in one case study attended Board of Health and Council meetings to address any enforcement related concerns directly (Ottawa). For more details on how by-laws were enforced in each case, please see the case descriptions (Appendix A – C).
- ***Find alternative, more cost-effective ways to promote the by-law in outdoor spaces***: Given the number of areas that are smoke-free as the result of a smoke-free outdoor space by-law, signage can be an extremely costly undertaking. In one case signage was paid for out of an existing budget (Barrie), in another case less costly decals

were developed and placed on garbage cans, and temporary signage for events were created (Hamilton). Key informants also suggested that signage could be developed after the by-law had been adopted and placed only in selected parks and other outdoor spaces. Locations could be determined based on compliance rates, with signage being posted in areas where compliance was found to be low. This strategy would be less costly, as fewer signs would need to be produced, and may lead to increased compliance in areas where this was a concern.

Recommendations for future action:

- Consider the fiscal environment in which the by-law is being proposed. If it is a period of fiscal restraint, highlight the ways in which the by-law can be adopted with little to no fiscal impact.
- Demonstrate through practice-based evidence from other municipalities or previous by-laws that the by-law will be self-enforcing.

3.2.5. Rely on internal and external partnerships and collaborations

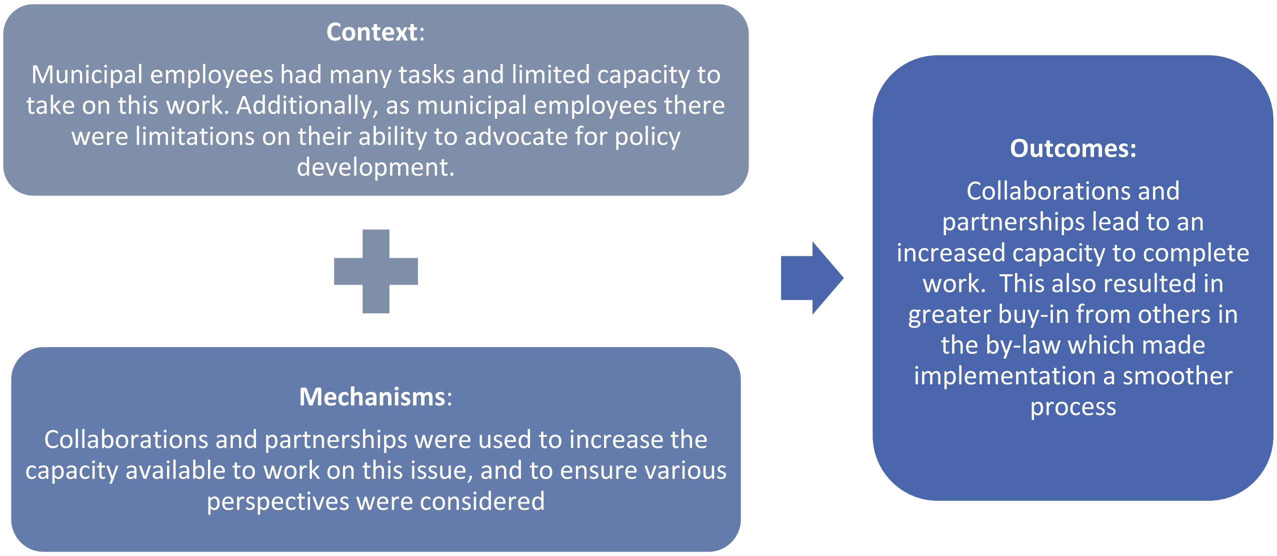

All three cases relied on partnerships with internal and external partners to move the policy agenda forward to ensure by-law adoption. Partnerships allowed for increased capacity to carry out work related to by-law development. Additionally, municipal staff cannot complete any work that could be perceived as advocacy. External partnerships can allow for advocacy activities to occur within the process. Partnerships were developed with the following groups:

Community advocacy groups and NGOs: Partnerships were formed between community advocacy groups and public health and between the groups themselves. In Barrie and Hamilton, partnerships were formed with NGOs to allow for information to be shared with decision makers. These partnerships were minimal, but provided an opportunity for NGOs to speak at decision maker meetings on the value of the proposed by-law. The partnerships and roles of these groups were strong in the City of Ottawa. The OCSH formed partnerships with various other community NGOs to ensure all individuals were on the same page about the type of by-law they would like to advocate for. Additionally, OCSH worked with public health to promote the need for a smoke-free space by-law to decision-makers. These partnerships were critical as the OCSH was able to advocate for smoke-free spaces in a way that municipal staff could not.

***Other municipal committees*:** Identifying other groups within the municipality working on related issues may allow for shared projects and additional support. For example, partnering with the Clean City Liaison Committee in Hamilton that led a cigarette butt cleanup project allowed public health practitioners to understand where tobacco-related litter was most problematic in the city.

Other municipal staff: Partnerships with other municipal departments such as parks and recreation, by-law, legal, and Tourism, was found to be critical to the development of the by-law. Given the scope of the by-law, employees from various departments were impacted. Having them on board from the by-law development stage led to greater buy-in and helped to improve the implementation work to be done. As a key informant noted, *"having everybody [engaged] was really essential and helped inform our decision-making and the directions we recommended and have helped forge ongoing cooperation and relationships as we move through the first year [of implementation]"* (Hamilton).

Public Health Practitioners: In Ottawa and Hamilton, public health took the lead on by-law development. In Barrie, the municipality took the lead on the by-law development and reached out to the health unit for support with the process. The health unit noted that the municipality *"knew we were a stakeholder in that they knew they could rely on us to support them and get them going forward, that we could get them the facts and rationale and answer questions*". The municipality reached out to the public health unit for support with enforcement. This was not possible due to capacity constraints, but the health unit did provide support and advice to the municipality to support by-law development and help them understand how the enforcement of the by-law would occur.

Recommendations for future action:

- Involve internal partners in the by-law development process from the outset, as it improves buy-in, ensures the by-law fits within other municipal partner's mandates and may make implementation easier.
- Partner with external groups that have an interest in smoke-free outdoor spaces to allow for greater advocacy work. Encourage these groups to speak with decision makers and attend Board of Health and Council meetings to make deputations.

3.2.6. Frame issues so they are of interest to a wide variety of stakeholders

Context:

There was an understanding that tobacco use is harmful, but there was also some concern about how far tobacco control policies should go in the name of protecting health.

+

Mechanisms:

Arguments for by-law development and the by-law's goals and objectives were framed around issues that went beyond public health (e.g., a productive workforce, improved community image, reduce litter, etc.).

Outcomes:

This broad framing led to increased support for smoke-free outdoor spaces. Careful framing can lead to greater buy-in from various municipal departments and groups as they can see how it fits with their mandates.

Following the identification of the development of smoke-free outdoor spaces as a priority area of interest, policy entrepreneurs and individuals working on the by-law development were tasked to identify the goals and objectives of the policy and to communicate the need for the policy to various groups and partners. Key informants from two of the three cases highlighted that it was important to frame the issue so it would be seen as relevant to many municipal departments, not only to public health. As one key informant noted: *"So, things around health, you know, we would re-frame (it) over time (to): we will see a change in the overall exposure and smoking rate, if the evidence is accurate, and that will only lead to a more productive and healthy community"* (Hamilton). A key strength of the partnerships that were formed with others within the municipality (as highlighted in section 3.2.5) was that it allowed for the issue to be viewed from more than just a public health perspective. As one key informant noted:

> *…it's not enough to say second-hand smoke is bad. I think that message has saturated itself with decision-makers. They get it. And they just—they think "Well yeah, you're the Public Health Department. You're getting paid to see advances in this area. Yeah, yeah, yeah." Right? I think…we, as a community, need to re-frame the argument so that the decision-makers understand the issue in the context of the world in which they find themselves. So, their primary motivators these days are things like economic liability, productivity, customer service. So if we can re-frame some of these issues in that context, I think it resonates a little more clearly for the decision-makers (Hamilton)*

Though only two of the three cases explicitly highlighted the importance of framing the issues so they were relevant to various stakeholders, when looking at how the issues were framed, all three cases did frame their arguments in a way that would allow various groups to identify with the concerns. Key informants framed the need for smoke-free spaces based on the following:

- Using language that was familiar to City Council – for example looking at customer service based arguments for smoke-free outdoor spaces (Hamilton)
- Emphasizing the environmental impact of tobacco use, and the potential benefits of smoke-free spaces for reducing tobacco-related litter (Hamilton, Barrie, Ottawa)
- Protecting individuals from exposure to SHS (Hamilton, Barrie, Ottawa)
- Assisting with cessation – smoke-free outdoor spaces reduce the number of opportunities for individuals to smoke and therefore can encourage individuals to quit (Barrie, Ottawa). Cessation supports can be offered in conjunction with the by-law being introduced (Ottawa).
- Potential to lead to a reduced smoking rate (Hamilton, Ottawa, Barrie) which could lead to a healthier and more productive community (Hamilton)
- Emphasizing that tobacco and healthy living do not go "hand in hand" and thus areas that are designated as being for sport activities should be smoke-free (Barrie, Hamilton)
- Levelling the playing field – some restaurants, parks and sport teams have adopted voluntary smoke-free policies and other spaces have already been mandated to be smoke-free (schools), creating a broad policy will create a more level playing field for everyone to work on (Hamilton, Ottawa)
- The importance of creating smoke-free spaces to reduce social exposure to tobacco use (Barrie, Hamilton, Ottawa)

Overall it was noted that the most important thing when framing the arguments for smoke-free outdoor spaces is to do so in a way that many individuals can identify with a reason to support the development, not just those with an interest in tobacco control or public health. Working with internal and external partners to frame the arguments is one way to ensure that they are relevant for a large audience.

Recommendations for future action:

- Involve internal and external partners in the discussions around how the goals and objectives of a by-law, and the messaging surrounding the by-law should be framed.
- Ensure the messages are relevant not only to those in public health, but more broadly to the municipality and community as a whole.

3.2.7. Highlight opportunities for innovation

Context:

Municipalities had been identified as being innovative in the past due to smoke-free indoor space work. There was a sense of a continued desire to be seen as innovative and to adopt by-laws that were gold standards.

+

Mechanisms:

Opportunities for municipalities to take on a leadership role and to become innovators in the area of smoke-free outdoor spaces were highlighted; by-law options that were innovative and moved the gold standard forward were highlighted.

→

Outcomes:

Interest in moving forward with by-law development was seen, and there was a clear desire to push for a comprehensive by-law that includes new and innovative components.

The concept of innovation and of becoming an innovator was identified as being critical in all three cases. The desire to be seen as innovators and to move the field of smoke-free outdoor spaces forward was a mechanism that motivated interest in and action for by-law development and underpinned many of the other mechanisms highlighted in this report.

Key informants from each municipality highlighted the importance of innovation to their cities. Key informants from SMDHU noted that decision makers in Barrie may have identified moving forward with smoke-free outdoor spaces as an opportunity to be *"seen as a leader"* and to continue their emphasis on developing a city that is a *"clean city that is a healthy place to live*". In Hamilton, it was highlighted that Hamilton's vision is to be the *"best place in Canada to raise a child*" and this desire to be innovative can be used to promote the need for by-law development. Additionally, decision makers in Hamilton highlighted the desire to understand how they could make an impact in the area of tobacco control following the implementation of the SFOA at the provincial level, highlighting that they wanted to continue innovation in that area. Key informants from Ottawa spoke of how Ottawa had been a leader in the past when it came to smoke-free spaces and that there was a desire for them to remain in a leadership position and thus they needed to take action on smoke-free outdoor spaces. They noted, *"the idea here is if we're going to do this, we're going to do this big. And provide as much protection to Ottawa residents as possible*".

The push for innovation and the desire to be innovators was identified in all three cases as a reason for moving forward with by-law development and a reason for pushing for a comprehensive by-law. In each of the cases studied, this meant that the municipality pushed beyond what others had adopted and found ways to develop a by-law that could be considered gold standard at the time. Barrie included beaches in their by-law, Hamilton included multiple

forms of tobacco use including the use of hookahs, and Ottawa included all municipal property as well as restaurant patios.

Being innovative in the by-law development process was not only seen as being critical for the municipalities themselves, but also for the province as a whole. Creating innovative by-laws can change the gold standard encouraging other municipalities to reach that level or push beyond it. Additionally, it was noted that the province likely wouldn't move forward with a provincial ban until they saw a clear movement from the ground. As with the first phase of by-law development where numerous municipalities passed local by-laws prior to the implementation of the SFOA at the provincial level, the continuous innovation at the municipal level in regards to smoke-free outdoor spaces is important for encouraging the move towards provincial legislation.

Recommendations for future action:

- Find opportunities for the municipality to be innovative, and highlight these to the individual municipalities. This may help encourage the development of a comprehensive by-law that breaks new ground for smoke-free spaces.
- Frame the issue of smoke-free outdoor spaces as an opportunity for a municipality to be innovators who may be setting potential benchmarks for others to emulate. This notion that the municipality can be leaders and can stay ahead of the curve can create motivation among decision makers to move forward with smoke-free outdoor space by-laws.

3.2.8. Create and take advantage of policy windows

Context:

There are many different issues that are of interest to decision makers at any given time. To create action in an area of interest, it was critical to wait for or create a "policy window" - an opportunity when a problem is recognized, there is a solution available and there is political will to move forward.

Mechanisms:

Policy entrepreneurs and others ensured decision makers were aware of the issue of smoke-free outdoor spaces and the need for a by-law. When opportunities to move forward with by-law development were presented, they ensured that decision makers had the information and support they needed to move forward.

Outcomes:

Political will for by-law development was present, decision makers were aware of the issue of smoke-free outdoor spaces and the solution to the problem was accepted allowing for by-law development.

Policy windows occur, according to Kingdon (2003) when the problem, policy and politics stream converge. This is a point in time when "*a problem is recognized, a solution is developed and available in the policy community, a political change makes it the right time for policy change, and potential constraints are not severe*" (Kingdon, 2003). In each of the three cases studied, a clear policy window opened allowing for the development of smoke-free outdoor spaces. Many of the mechanisms highlighted throughout this report contributed to the opening of the policy window. Across

the three cases a desire to continue to build the momentum that was generated from the individual municipalities' smoke-free indoor-space by-laws and the SFOA and a desire to be innovators in the area of smoke-free outdoor spaces and to stay ahead of the curve in regards to second-hand smoke pushed the desire to open a policy window in this area. The mechanisms highlighted in this report all worked to either help open the policy window, or to encourage by-law development once the policy window had opened. Being aware of opportunities for when by-laws can be created and taking advantage of these windows of opportunity is critically important. The policy window that opened in each case is described below. More information on the specifics of each case are outlined in the case descriptions found in Appendices A – C.

Barrie: The City of Barrie began looking at the issue of smoke-free outdoor spaces following the passage of the SFOA at the provincial level. The move towards a smoke-free outdoor space by-law was Councillor-led, with the initial push for the by-law coming from Barrie City Council in September 2007. City Council adopted a motion requesting the City Clerk's office to investigate the feasibility of prohibiting smoking within nine metres of entrances and exits to city facilities and children's playgrounds and to report back to the General Committee with their findings. Key informants from SMDHU suggested that this push for smoke-free outdoor spaces may have stemmed from the fact that the dangers of second-hand smoke (SHS) exposure had "*become something that was accepted*", and the sense that *"okay, we've got a bit started, what can we do to keep this ball rolling and protect our community?"* (SMDHU Staff). Some Barrie City Councillors sit on the Board of Health at the regional level, and it was noted by a key informant from SMDHU that discussions at that table about the need to move beyond the SFOA may have encouraged local level action. Once City Council identified a desire to look at smoke-free outdoor spaces, public health staff worked to ensure that the Council had the information and support they needed to move forward with developing a comprehensive by-law.

Hamilton: The SFOA had passed at the provincial level and as the SFOA was more comprehensive than Hamilton's indoor smoke-free space by-laws it moved tobacco control in the city forward. At that time, the City of Hamilton's Board of Health identified an interest in understanding what decision making power they still had in the area of tobacco control, and in considering the next steps they could take to make an impact in the area. To facilitate this work, the Board of Health directed Hamilton Public Health Services (HPHS) to identify action items and a series of recommendations regarding the city's jurisdictional authority to impact tobacco control for the betterment of residents and visitors. This was a window of opportunity for HPHS to make an argument for smoke-free outdoor spaces and the need to move that to the front of the policy agenda. HPHS staff submitted a report and presentation to the Board of Health regarding next steps for tobacco control in Hamilton, and this included a recommendation to move forward with outdoor space by-law work. The Board approved moving forward with the issue in March of 2008, and requested a review of the by-law in order to assess the feasibility of creating tobacco-free parks, playgrounds and beaches within the municipality. This created the opportunity for by-law development in the City of Hamilton.

Ottawa: Work advocating for smoke-free outdoor spaces in Ottawa began before a policy window of opportunity opened in the municipality. The OCSH felt that Ottawa was starting to lag behind others in the area of smoke-free outdoor spaces. Other municipalities in Ontario had begun to adopt by-laws creating smoke-free outdoor spaces, and the OCSH felt it was time for Ottawa to do the same. As a result, OCSH took a lead role in advocating for the need for smoke-free outdoor space by-laws and began promoting the benefits of and reasons for smoke-free spaces to the community and decision-makers. OPH had also identified smoke-free outdoor spaces as a priority area of work. Since the initial smoke-free space by-law was passed in 2001, the City of Ottawa has worked to *"continue to provide smoke-free spaces to citizens"* (OPH Staff). OPH knew, however, to move forward with taking a regulatory

approach to these issues they would need the *"political will of city counselors to back it"* (OPH Staff). While waiting for the political appetite for by-law development, they focused on voluntary policy development and education.

In September 2011, an Ottawa City Councillor put forward a motion requesting the Board of Health review and report back on the public health value of designating beaches, parks, and patios smoke-free until 8:00pm. As a result of this motion, the Board of Health directed OPH staff to conduct a comprehensive study and develop recommendations to move forward. This was the window of opportunity that the champions in Ottawa had been waiting for and working towards, and this provided an opportunity to move forward with by-law development and to push for a comprehensive by-law to be implemented.

All three cases highlight how policy windows can open in different ways in different contexts. A common theme, however, is the importance of being aware of these types of opportunities and using them to bring smoke-free outdoor spaces to the front of the policy making agenda.

Recommendations for future action:

- Maintain an understanding of decision makers' readiness to address tobacco related issues, and opportunities to move smoke-free outdoor spaces to the front of the agenda.

3.3. Mechanisms related to by-law implementation

Once the municipalities had adopted a smoke-free outdoor space by-law, policy entrepreneurs and others remained engaged in the implementation of the by-law. A strong implementation process was critical to ensuring that the by-law would be successful. Mechanisms that were found to be critical to the implementation of the smoke-free outdoor space by-law are highlighted in the following sections. As noted earlier, many of the mechanisms that fired in the by-law development phase continued to play a role in the by-law implementation phase as well, and therefore should still be considered when planning by-law implementation.

3.3.1. Create a shared vision and understanding among municipal employees

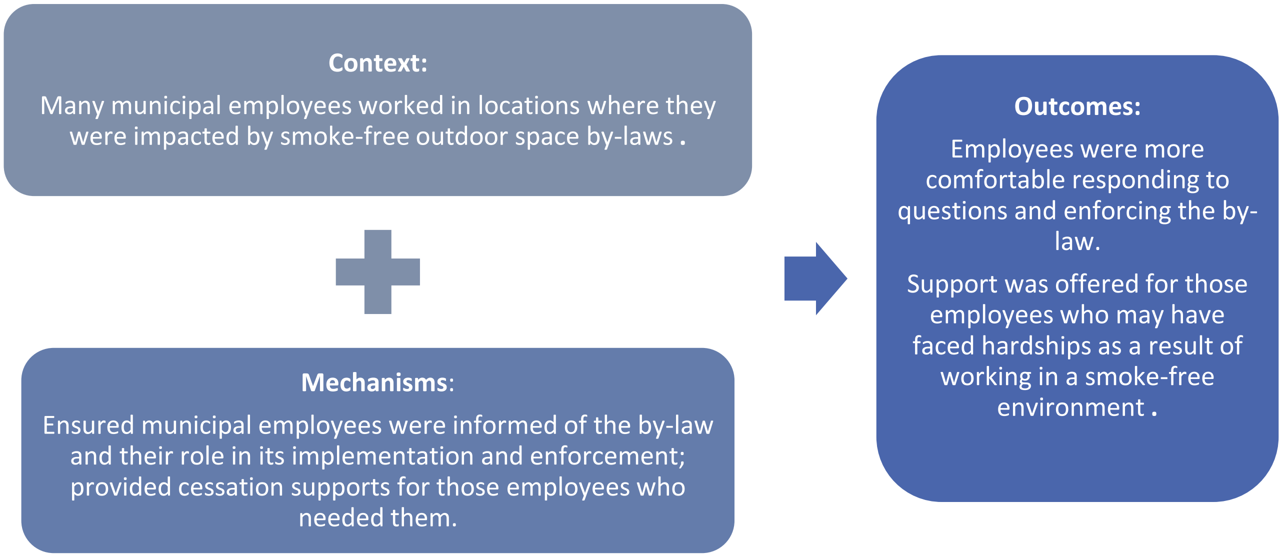

It was acknowledged in all three cases that implementing a smoke-free outdoor space by-law could significantly impact municipal employees working in the affected areas. It was noted that employees may be required to respond

to concerns or questions about the by-law and its implications, they may be required to deal with non-compliance, and if they themselves smoke, they would need to find a way to manage their smoking behaviours given the understanding that they can no longer smoke on municipal property. Addressing these potential concerns from the outset was identified as being critical to the success of the by-law. Key informants from each case reported that they used various strategies to keep municipal staff informed, and to assist with the hardships that they may face as a result of this by-law. Strategies included:

- Electronic correspondence to all city employees regarding the by-law (Hamilton)
- Staff education sessions (Ottawa, Hamilton, Barrie)
- Cessation supports for employees (Ottawa, Barrie, Hamilton)
- Training on the updated by-laws and staff roles in its implementation and enforcement (Ottawa, Hamilton)
- Provision of materials to support municipal staff who may be asked to respond to complaints regarding an individual smoking in a designated non-smoking area (Hamilton)

Recommendations for future action:

- Consider how municipal employees will be impacted as a result of a new or revised by-law.
- Determine ways to minimize the potential impacts – cessation supports for employees, education on the by-law, provisions of resources to aid in enforcement.

3.3.2. Develop a strong communication strategy

Context:

There was limited funding available to support proactive enforcement of by-laws. It was understood from past by-law work that if the community is aware of a by-law, compliance will increase.

Mechanisms:

A phased approach to by-law enforcement was taken beginning with an educational phase.

Various communication strategies (paid and earned media, in-person visits, presence at community events) were utilized to ensure awareness of the smoke-free outdoor space by-law.

Outcomes:

Community members were aware of the smoke-free outdoor space by-law and how it applied to them. Initial compliance rates were good.

When introducing the smoke-free outdoor space by-laws, each community elected to take a phased-in approach to enforcement focusing on education and warnings for a set period of time before moving towards issuing tickets to those who are not in compliance with the by-law. This phased in approach ensured that there was time for individuals to become aware of the by-law and to alter their behaviour accordingly. To ensure community members were aware of the new by-law, a key component of the enforcement strategy in all three cases was to communicate with the public to ensure they were aware of the new by-law. It was thought that by increasing communications about the by-

law, self-enforcement and compliance would also increase. Activities undertaken by individuals from the three cases to promote the by-law included:

- In-person visits to all hospitality businesses impacted by the by-law amendments (those with a patio) by public health and enforcement staff (Ottawa)
- Flyers announcing the new smoke-free outdoor space regulations (Ottawa)
- Advertising campaign including television, bus, newspaper and/or radio ads (Barrie, Ottawa, Hamilton)
- Social marketing campaigns including twitter posts and Facebook ads (Ottawa, Hamilton)
- Temporary and permanent signage (Ottawa, Barrie & Hamilton)
- Training for event organizers about the smoke-free space by-law and its impact on their events (Ottawa)
- Earned media through press releases and other tactics (Ottawa, Barrie, Hamilton)
- Including information in existing municipal publications (e.g., tourism guides, recreation guides, politicians newsletters; Barrie & Hamilton)
- Meetings with sport organizations and provision of promotional materials (e.g., soccer balls, lanyards, water bottles; Hamilton)
- Booths at community festivals and events (Hamilton, Ottawa)

Copies of various communication tools are included in each of the case descriptions (Appendices A – C). Having the resources to communicate to residents that the by-law had come into effect was recognized as a mechanism that led to success in implementation and relaying the message. For example, in Hamilton staff relayed the message of the by-law to individuals entering events, and heard that most people were aware of the by-law. It was recognized that this strong, initial communication campaign eased enforcement concerns, as it was indicated that awareness of the by-law generally translated into compliance.

Recommendations for future action:

- Ensure a comprehensive communications plan is developed prior to and following by-law development. Awareness of the by-law may increase compliance.
- When resources to communicate the message are limited, focusing on earned media and taking advantage of existing municipal publications are two ways to communicate the message with limited costs.

4.0 Conclusions

In Ontario, much has been done to protect the health of citizens by reducing the physical and social exposure to tobacco smoke indoors through both provincial and municipal legislation. This second phase of smoke-free space by-law development which focuses on the need for smoke-free outdoor spaces continues this work and provides additional protection for citizens. The key learnings from the work completed by Barrie, Hamilton and Ottawa outlined in this report may be beneficial for others who are hoping to create or amend smoke-free outdoor space by-laws in their own municipalities to keep in mind. Underpinning many of the mechanisms highlighted throughout the report was a desire on the part of the municipality to continue a history of innovation, to identify opportunities to be leaders in the field of smoke-free spaces and the importance of policy entrepreneurs. The desire for innovation provided an impetus for decision makers to move forward with by-law development and influenced the scope of by-laws created. Building on the work that had been completed in the campaign for smoke-free indoor space by-laws provided an opportunity to highlight how the municipality had been innovative in the past, and how it could continue this legacy. This past work also worked to 'soften up' the community and decision makers, increasing their willingness to move forward with by-law development. None of the work, however, could have occurred without policy

entrepreneurs. These individuals created opportunity for by-law development, pushed the agenda forward and were the ones who put most of the critical mechanisms into play. Policy entrepreneurs came from a variety of fields and backgrounds, and different individuals took on a leadership role in each municipality. A common thread across the municipalities, however, was the role public health practitioners played in ensuring the implementation of the by-law. Collaborations between public health and other internal and external partners allowed for increased capacity for by-law development work, and ensured the issue of smoke-free outdoor spaces was framed in a way that would be understood by others in the municipality, and would be seen as an issue of interest. This collaborative approach continued into the by-law implementation phases where it was noted that it is critical to ensure that all municipal staff are on board with the by-law, understanding their role in its implementation and enforcement, and feeling supported in working through issues the by-law may pose to their work day, for example, how they will deal with tobacco cravings while on municipal smoke-free property.

While many Ontarians are protected from exposure to SHS in outdoor spaces, there are many who are not. If smoke-free outdoor spaces are to become the norm, there remains a need to push for smoke-free outdoor space by-laws to be passed in municipalities where they are not yet in place, and to ensure all new and existing by-laws are comprehensive. Multi-disciplinary teams of City Councillors, municipal staff, public health practitioners, advocates and others can work together to move this agenda forward and continue to protect the health of Ontarians.

References

Alesci, N. L, Forster, J. L., & Blaine, T. (2003). Smoking visibility, perceived acceptability, and frequency in various locations among youth and adults. *Preventive Medicine, 36*, 272-281.

Bloch, M., & Shopland, D. (2000). Outdoor smoking bans: more than meets the eye. *Tobacco Control, 9*, 99.

Cameron, M., Brennan, E., Durkin, S., Borland, R., Travers, M. J., Hyland, A., Spittal, M. J., & Wakefield, M. A. (2010). Second hand smoke exposure (PM2.5) in outdoor dining areas and its correlates. *Tobacco Control, 19,* 19-23.

Ministry of Health and Long-Term Care, Government of Ontario. (2011). *The act in brief: Smoke-Free Ontario Act – protecting Ontarians.* Retrieved from http://www.mhp.gov.on.ca/en/smoke-free/legislation/in-brief.asp

Ipsos Ried. (2011). *Majority of Ontarians support Smoke Free Ontario Act and further tobacco control measures*. Retrieved 25 Sept 2012 from http://www.newswire.ca/en/story/774321/majority-of-ontarians-support-smoke-free-ontario-act-and-further-tobacco-control-measures

Kaufman, P., Zhang, B., Bondy, S. J., Klepeis, N., & Ferrence, R. (2011). Not just 'a few wisps': real-time measurement of tobacco smoke at entrances to office buildings*. Tobacco Control, 20*, 212 – 218.

Kennedy, R. D. (2010). *Smoke-free patios – a study of air quality on patios that permit or restrict smoking in the city of Ottawa*. Retrieved 30 Jan 2013 from http://www.smokefreeottawa.com/uploads/1/1/7/4/11742147/newsconferencebackgrounder.pdf

Kingdon, J. W. (2003). *Agendas, alternatives, and public policies (2nd ed.)*. New York: Longman.

Klepeis, N., Ott, W. R., & Switzer, P. (2007). Real time measurement of outdoor tobacco smoke particles. *Journal of the Air and Waste Management Association, 57*, 522 – 534.

Lippert, W. C., & Gustat, J. (2012). Clean Indoor Air Acts reduce the burden of adverse cardiovascular outcomes. *Public Health, 126(*2012), 279-285.

Mackay, D., Haw, S., Ayres, J. G., Fischbacher, C., & Pell, J. P. (2010). Smoke-free legislation and hospitalizations for childhood asthma. *New England Journal of Medicine, 363*(12), 1139-1145.

Maxwell, J. A. (2004). Using qualitative methods for causal explanation. *Field Methods, 16*(3), 243 – 264.

Meyers, D. G., Neuberger, J. S., & He, J. (2009). Cardiovascular effect of bans on smoking in public places: A systematic review and meta-analysis. *Journal of the American College of Cardiology, 54*(14), 1249-1255.

Mozaffarian, D., Afshin, A., Benowitz, N. L., Bittner, V., Daniels, S. R., Franch, H. A., et al. (2012). Population approaches to improve diet, physical activity and smoking habits: A scientific statement from the American Heart Association. *Circulation, 126*, 1514-1563.

Non-Smokers' Rights Association. (2012). *Non-Smokers' Rights Association smoke-free laws database.* Retrieved from http://www.nsra-adnf.ca/cms/smoke-free-laws-database.html

Nykiforuk, C., d'Avernas, J., Lovato, C., Manske, S., Perley, M., Smith, P., Viehbeck, S., & Filsinger, S. (2010). *Smoke-free spaces: lessons from a better practice review of a population health intervention.* Edmonton, AB: Centre for Health Promotion Studies, University of Alberta.

Ontario Campaign for Action on Tobacco. (2012). *Municipalities with 100% smoke-free bar/restaurant by-laws*. Retrieved 9 October 2012 from http://www.ocat.org/pdf/BylawsPopulation.pdf

Ontario Tobacco Research Unit. (2012). *Smoke-Free Ontario Strategy evaluation report*. Toronto, ON: Ontario Tobacco Research Unit.

Pawson, R., & Tilley, N. (1997). *Realistic evaluation.* London, United Kingdom: Sage.

Rehm, J., Balliunas, D., Brochu, S., Fischer, B., Gnam, W., Patra, J., Popova, S., Sarnocinska-Hart, A., & Taylor, B. (2006). *The costs of substance abuse in Canada 2002*. Ottawa, ON: Canadian Centre on Substance Abuse.

Smoke-Free Ontario – Scientific Advisory Committee. (2010*). Evidence to guide action: Comprehensive tobacco control in Ontario*. Toronto, ON: Ontario Agency for Health Protection and Promotion.

Stake, R. E. (2006). *Multiple case study analysis*. New York, NY: Guilford Press.

Statistics Canada. (2013). *Population and dwelling counts, for Canada, provinces and territories, and census subdivisions (municipalities), 2011 and 2006 censuses.* Retrieved from http://www12.statcan.gc.ca/census-recensement/2011/dp-pd/hlt-fst/pd-pl/Table-Tableau.cfm?LANG=Eng&T=302&PR=35&S=51&O=A&RPP=25

United States Department of Health and Human Services. (2006). *The health consequences of involuntary exposure to tobacco smoke: a report of the Surgeon General.* U.S. Department of Health and Human Services, Centers for Disease Control and Prevention, Coordinating Center for Health Promotion, National Center for Chronic Disease Prevention and Health Promotion, Office on Smoking and Health.

Wilson, L. M., Tang, E. A., Chander, G., Hutton, H. E., Odelola, O. A., Elf, J. L., et al. (2012). Impact of tobacco control interventions on smoking initiation, cessation, and prevalence: A systematic review. *Journal of Environmental and Public Health, 2012*, Article ID: 961724.

Yin, R. (2009). *Case study research design and methods, 4th edition*. Thousand Oaks, California: Sage Inc.
