# Supplementary figures and images for: A mixed methods evaluation of capturing and sharing practitioner experience for improving local tobacco control strategies
Source: Can J Public Health. 2018 Nov 19;110(1):103–13. doi: 10.17269/s41997-018-0153-3 (PMC6335370; doi:10.17269/s41997-018-0153-3)

# Logic model for PTCC Documentation of Practice - 07-20-2016

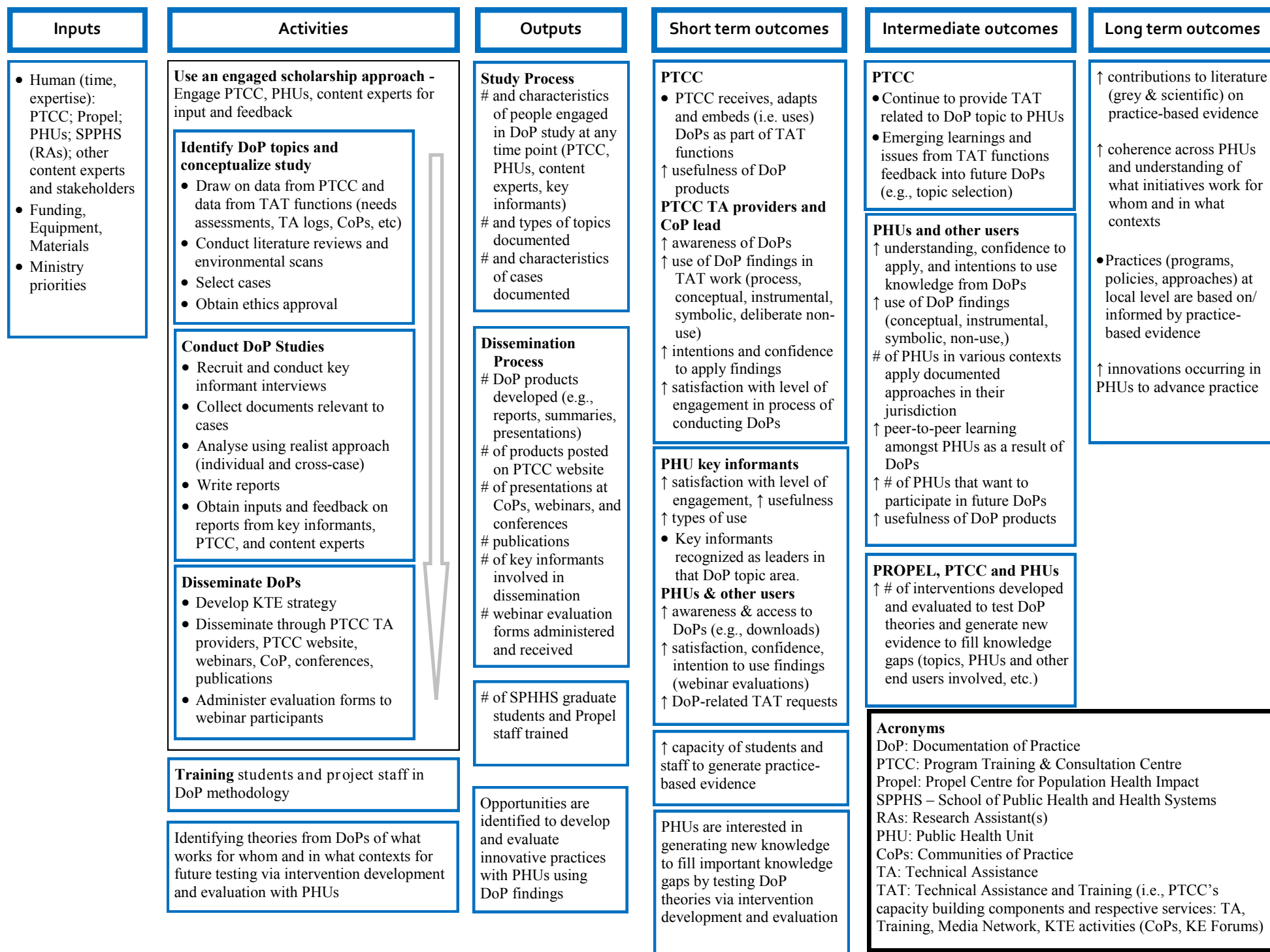

Supplement: Supplementary file 2 — (PDF 225 kb) [file 41997_2018_153_MOESM2_ESM.pdf]
